# Template-directed RNA polymerization and enhanced ribozyme catalysis inside membraneless compartments formed by coacervates

**DOI:** 10.1038/s41467-019-08353-4

**Published:** 2019-01-30

**Authors:** Raghav R. Poudyal, Rebecca M. Guth-Metzler, Andrew J. Veenis, Erica A. Frankel, Christine D. Keating, Philip C. Bevilacqua

**Affiliations:** 1 Department of Chemistry, The Pennsylvania State University, University Park, PA 16802 USA; 2 Center for RNA Molecular Biology, The Pennsylvania State University, University Park, PA 16802 USA; 3 Department of Biochemistry, Microbiology, and Molecular Biology, The Pennsylvania State University, University Park, PA 16802 USA; 4 Present Address: School of Chemistry and Biochemistry, Georgia Institute of Technology, Atlanta, GA 30332 USA; 5 Present Address: The Dow Chemical Company, 400 Arcola Road, Collegeville, PA 19426 USA

## Abstract

Membraneless compartments, such as complex coacervates, have been hypothesized as plausible prebiotic micro-compartments due to their ability to sequester RNA; however, their compatibility with essential RNA World chemistries is unclear. We show that such compartments can enhance key prebiotically-relevant RNA chemistries. We demonstrate that template-directed RNA polymerization is sensitive to polycation identity, with polydiallyldimethylammonium chloride (PDAC) outperforming poly(allylamine), poly(lysine), and poly(arginine) in polycation/RNA coacervates. Differences in RNA diffusion rates between PDAC/RNA and oligoarginine/RNA coacervates imply distinct biophysical environments. Template-directed RNA polymerization is relatively insensitive to Mg^2+^ concentration when performed in PDAC/RNA coacervates as compared to buffer, even enabling partial rescue of the reaction in the absence of magnesium. Finally, we show enhanced activities of multiple nucleic acid enzymes including two ribozymes and a deoxyribozyme, underscoring the generality of this approach, in which functional nucleic acids like aptamers and ribozymes, and in some cases key cosolutes localize within the coacervate microenvironments.

## Abstract (web summary)

Membraneless compartments have been theorized to be prebiotic micro-compartments as they spontaneously encapsulate RNA and proteins. Here, the authors report membraneless compartments can enhance RNA chemistries, affecting template directed RNA polymerization and stimulating nucleic acid enzymes.

## Introduction

The RNA world hypothesis posits roles for RNA as catalysts and carriers of genetic information on the primordial Earth^1,2^. Discovery of multiple natural and artificial ribozymes and aptamers has demonstrated RNA’s intrinsic ability to catalyze and regulate chemical reactions^3–9^. It is generally accepted that the transition from rich prebiotic chemistry to the earliest lifeforms must have included simpler compartments and protocells as intermediates to highly evolved compartmentalized systems consisting of genetic and catalytic polymers. Several lipid and fatty acid-containing membrane systems have been shown to facilitate compartmentalization^10,11^ and support template-directed polymerization of RNA^12^. However, permeability and transport in lipid-based vesicles is highly sensitive to the size of molecules, and especially unfavorable as the cargo becomes charged and larger^13^.

Liquid–liquid phase-separated (LLPS) systems have been hypothesized as model prebiotic compartments^14–16^ because of their ability to assemble spontaneously from a variety of oligomeric or polymeric components and to encapsulate otherwise dilute solutes, enabling local concentrations of potentially functional molecules such as nucleic acids at many orders of magnitude higher than is present in the overall solution^17^. LLPS also allows diffusion both within and between the compartment and the surrounding environment, even for large and charged molecules such as RNAs^18,19^. Complex coacervates, which arise from phase separation of oppositely charged polyions, have been hypothesized as model protocells, or alternate biophysical compartments for prebiotic reactions^15,20,21^. Membraneless compartments such as the nucleolus and RNP granules exist in modern cells and have their own biological functions^22–24^. Formation of these RNA and protein-rich intracellular liquid phases relies on not only charge–charge interactions, but also π–π stacking, H-bonding, cation–π, and dipole–dipole contacts^25^. It is possible that biological membraneless organelles may have arisen as simple abiogenic phase-separated systems such as complex coacervates.

The highly charged nature of RNA suggests that it could have participated in formation of complex coacervates with polycations on early-Earth, thus providing primitive compartments for functional RNAs such as ribozymes and RNA aptamers. Polycations such as polyamines have been shown to condense nucleic acids and form complex coacervates with nucleotides, RNA, and other anionic polymers^18,26,27^. High concentrations of nucleotides have been reported for coacervates containing polylysine and ATP^16^. Similarly, coacervates of poly(allylamine) (PAH) and ATP strongly concentrate RNA molecules, nucleotides, and Mg^2+^ ions^17^. Compartmentalization in aqueous two-phase systems consisting of the uncharged polymers polyethylene glycol (PEG) and dextran has been shown to enhance ribozyme catalysis due to increased local concentration^28^. However, RNA functions in complex coacervates, which have quite different composition and properties than aqueous two-phase systems, are largely unexplored. Importantly, RNA partitioning within complex coacervates generally involves ion-pairing interactions with polycationic components^17,18^, which may inhibit necessary RNA functions by disrupting native RNA structures. In fact, certain biological membraneless compartments have been shown to destabilize RNA duplexes^29^. The hammerhead ribozyme has recently been shown to retain some activity inside polylysine/carboxymethyldextran complex coacervates, with rates 60-fold slower than in the absence of coacervates^30^.

In this report, we study the effect of coacervate composition on template-directed polymerization of RNA, and explored RNA aptamer and ribozyme activities within membraneless compartments formed by complex coacervates. We show that the identity of cationic polymers in the coacervates significantly affects template-directed polymerization of RNA molecules. We first demonstrate that coacervates made with specific polyamines can inhibit template-directed RNA polymerization, while some enhance the reaction at sub-optimal Mg^2+^ levels. The polyamines used in this study have varying charge-densities and propensity to interact with nucleic acids. Poly(allylamine) (PAH-260) has a high charge density with ~17 charges per kDa (see Reagents and synthesis in Methods), whereas oligoarginine (R_10_), polydiallyldimethylammonium chloride (PDAC-53), and oligolysine (K_10_) have much lower charge densities at ~6.2, 5.2, and 6.3 charges per kDa, respectively. We show that fluorescent RNA aptamers remain bright inside coacervates, thus maintaining their native fold. Finally, we demonstrate that RNA-cleavage reactions catalyzed by multiple ribozymes and a DNAzyme are enhanced in the presence of coacervates. Thus, depending on their chemical composition, complex coacervates could have not only supported RNA copying and RNA aptamer–ligand binding, but also enhanced ribozyme activity on early-Earth while efficiently compartmentalizing RNA and other biomolecules from bulk solutions.

## Results

### Impact of polyamines on template-directed RNA polymerization

**Fig. 1:**
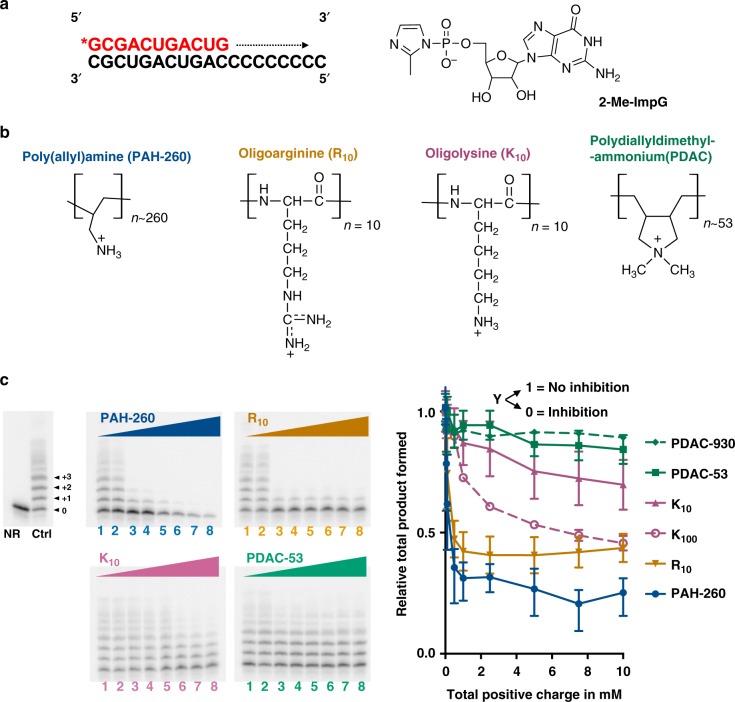
Effect of polyamines on template-directed polymerization. **a** Primer (red)-template (black) complex used for template-directed polymerization using guanosine 5′-(phosphor)-2-methylimidazolide (2-Me-ImpG). **b** Structures of polyamines used. **c** Representative denaturing polyacrylamide gel images show extension of ^32^P-labeled primer. Lane Ctrl indicates reaction without any cationic polymer and NR indicates no reaction in the absence of monomer, lanes 1–8 contain 0.050, 0.10, 0.50, 1.0, 2.5, 5.0, 7.5, and 10 mM total positive charge from PAH-260 (blue), R_10_ (orange), K_10_ (dark pink), and PDAC-53 (green). Reactions were carried out for 5 h and contained < 50 nM 5ʹ-end-labeled primer and 1 µM template in 25 mM Tris–HCl pH 8.0 and 5 mM MgCl_2_. Fraction converted to product was normalized to the yield in the absence of any cation ctrl. Error bars represent S.E.M. from three independent experiments. Uncropped gel images are shown in Supplementary Figure 14a

We first sought to understand how the chemical identity of polyamines that form complex coacervates affects template-directed polymerization of RNA with activated nucleotides (Fig. 1a). We carried out template-directed primer extension reactions at pH 8.0 in the presence of different polyamines (Fig. 1b) that are largely protonated at this pH. To study the effect of polyamines themselves, we performed the reactions in the absence of other polyions besides the RNA primer–template complex (i.e., no other polyanions to induce coacervation). Template-directed polymerization of RNA was assessed by the electrophoretic mobility shifts (+1, +2, and +3 etc.) arising from incorporation of nucleotides (Fig. 1c). We saw significant inhibition in the total amount of product formed by primer extension after 5 h in the presence of ≥500 µM total positive charge from PAH-260 (Fig. 1c). Similarly, oligoarginine (R_10_) also showed inhibition at ≥500 µM total positive charge, with product yield reduced by more than half compared to reactions in the absence of any cationic polymers. Although R_10_ has lower charge density than PAH-260 (~6.2 vs 17 charge/kDa), the level of inhibition is similar for these polycations. Recently, 10-mer oligoarginine was reported to inhibit ribozyme-catalyzed RNA polymerization at 10 µM^31^; suggesting that inhibition of RNA chemistries by oligoarginine may be a prevalent phenomenon. Ribozyme-catalyzed RNA polymerization has been reported to be enhanced in the presence of 10-mer oligolysine and other heteropeptides derived from the ribosomal core proteins^31^; however, we did not observe any significant enhancement or inhibition of template-directed polymerization of RNA in the presence of a 10-mer oligolysine (K_10_), which has a charge density of 6.3 charge/kDa. Template-directed polymerization was however inhibited by larger 100-mer polylysine (K_100_), as well as protamine, which is a natural arginine-rich protein (~5 charge/kDa) (Supplementary Figure 1a, b left). These findings suggest that charge density may not be the primary driver in inhibition of template-directed polymerization.

To further characterize the role of polyamines in inhibition of template-directed polymerization, we tested the effects of polyallyldiammonium chloride (PDAC), a quaternary amine that has been reported to form weaker/more labile ion-pairing interactions as compared to PAH^26^. We reasoned that weaker interactions between the polycationic component and the polyanionic RNA primer–template complex due to buried positive charge may reduce interference with template-directed polymerization. Strikingly, template-directed polymerization was maintained up to 10 mM total positive charge of PDAC-53 (Fig. 1c). Furthermore, the reaction was unaffected in the presence of PDAC with ~930 monomers (PDAC-930) (Supplementary Figure 1b, right). These results suggest that inhibition of template-directed polymerization by polyamines may thus be alleviated in cases where the amines are conformationally locked and the positive charge resulting from the protonation of nitrogens is shielded by other functional groups, preventing strong and interfering interactions with the nucleic acid. The difference in inhibition between K_10_ and K_100_ may be due to increased multivalency and flexibility of K_100_ compared to K_10._ No significant difference was seen in the amount of 2-Me-ImpG remaining after 5 h of incubation in the presence of different polycations, suggesting that the observed inhibition was not due to polyamines reacting with 2-Me-ImpG (Supplementary Figure 1c). These results are in line with previous observations where only about 12% of 2-Me-ImpG degraded in the presence of spermine in 18 days at pH 7.0^32^. Microscopy images revealed that at 1 mM total charge concentration, PAH and R_10_ are able to form phase-separated droplets with 10 mM 2-Me-ImpG even in the absence of longer polyanions (Supplementary Figure 2). These data are consistent with the greater propensity of these polyamines for ion-pairing interactions, not only with the primer/template complex, but even with nucleotide monomers. To study the effect of coacervation, we focused our attention to PDAC-53 and K_10_, which did not form any detectable droplets with the nucleotide monomer.

### PDAC coacervates support template-directed polymerization

We next explored the suitability of different coacervates for template-directed RNA polymerization. An 11-mer polyA RNA molecule (rA_11_) was used as the polyanion to drive phase separation of PDAC-53, K_10,_ or R_10_ at 10 mM, 1:1 charge balance (Supplementary Figure 3a, top panels). Fluorescently labeled RNA primer pre-annealed to the template was used to verify selective RNA partitioning inside the coacervate phase (Supplementary Figure 3a, bottom panels). To evaluate the concentrations of RNA primer inside the coacervate phase, fluorescence in the droplets was compared to a standard curve made in buffer (Supplementary Figure 3b). When just 0.5 µM of the primer complexed with 0.75 µM template was added to the bulk, the concentrations of primer inside coacervate phases were calculated to be around 78 ± 6, 30 ± 1, and 56 ± 12 µM for PDAC-53, K_10_, and R_10_ coacervates, respectively, indicating that all three coacervate systems strongly partition RNA inside the condensed phase.

**Fig. 2:**
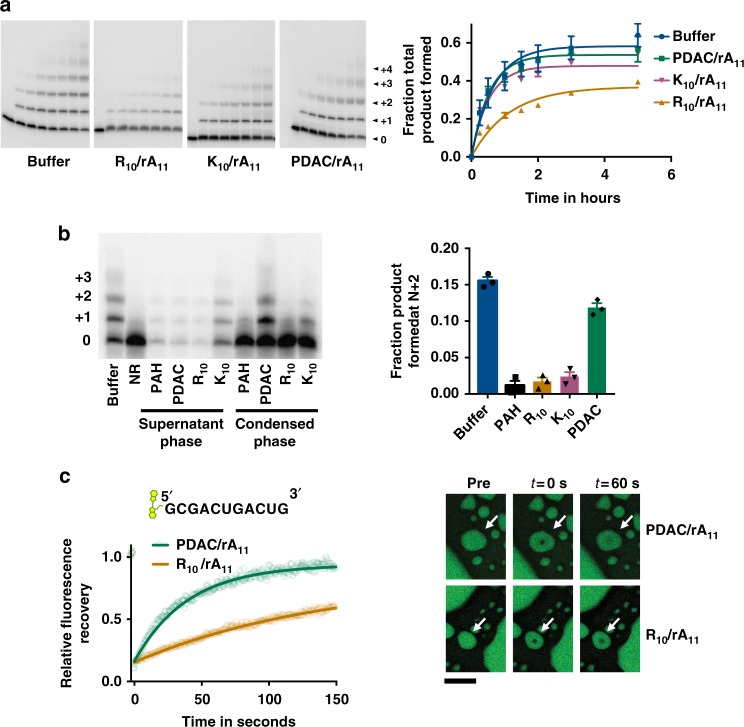
Coacervates of PDAC/rA_11_ RNA support template-directed polymerization. **a** Template-directed polymerization reactions in the presence of coacervates. Coacervates were formed by adding respective polycations (10 mM total positive charge) to solutions containing 10 mM 2-me-ImpG and 7.5 mM total negative charge from rA_11_ in 5 mM Mg and 25 mM Tris–HCl pH 8.0. Reactions were initiated by adding pre-annealed 5′-end-labeled RNA primer and unlabeled template, and incubated at room temperature and time points were taken at 0 min, 15 min, 30 min, 1 h, 1.5 h, 2 h, 3 h, and 5 h. Samples were separated by denaturing PAGE. Total product formed was calculated by quantifying all the visible bands in the given lane. Data were fit to first order exponential. Error bars represent S.E.M from three independent experiments. **b** Reactions were initiated as **a** except the Mg concentration was decreased to 1 mM to slow the reactions. Reactions were immediately centrifuged at 14,000×*g* after initiation. The bulk phase was then separated from the condensed phase and the two phases were allowed to react separately for 5 h. Quantifications of product yields are shown for condensed phases. Error bars represent S.E.M. from three independent experiments. **c** FRAP recovery curves for Cy3-labeled RNA primer in PDAC/rA_11_ and R_10_/rA_11_ coacervates at 10 mM charge-balanced condition. Shown as best fits to Eq. (2) (five independent trials). Representative images of PDAC/rA_11_ (top) and R_10_/rA_11_ (bottom). Scale bar is 5 µm. Uncropped gel images are shown in Supplementary Figure 14b, c

Next, we compared template-directed polymerization of RNA in the presence of coacervates made using PDAC-53, K_10_, or R_10_ as the polycation and rA_11_ as the polyanion. 10 mM total positive charge from each of the cationic polymers and 7.5 mM total negative charge from rA_11_ was used to drive coacervation. This charge imbalance was chosen to facilitate coacervate uptake of 2-Me-ImpG, which has one negative charge at reaction conditions. The fraction of primer converted to polymerized products by template-directed polymerization in PDAC-53/rA_11_ and K_10_ /rA_11_ coacervates were similar to buffer at about 50% after 5 h, but yields were lower for R_10_/rA_11_ coacervates, at about 30% after 5 h (Fig. 2a). Microscopy experiments revealed that PDAC-53/rA_11_ coacervates remain stable over the time course of template-directed polymerization of RNA (Supplementary Figure 4a).

To demonstrate that the template-directed polymerization reaction occurs inside complex coacervates and not the dilute phase, we centrifuged the reactions and separated the bulk supernatant and the condensed phase. Reactions were allowed to proceed independently in the separated phases. Quantification of radiolabeled signal from the gel image for both coacervates and supernatant revealed that >90% of the primer partitioned to the condensed phase for PDAC-53 and R_10_ coacervates, while ~85% and ~65% of the RNA remained in the condensed phase for PAH-260 and K_10_, respectively (Supplementary Figure 4b). Furthermore, higher bands (N + 2 and N + 3) were observed only for coacervates containing PDAC-53 and rA_11_ indicating significant template-directed RNA polymerization (Fig. 2b). These data show that among the coacervate compositions tested, only the PDAC-53/rA_11_ coacervates are suitable to yield longer RNA polymers from template-directed RNA polymerization. It should be noted that we observed addition of up to two nucleotides in the supernatant phase of R_10_/rA_11_ coacervates, suggesting that polymerized bands in Fig. 2a for this condition is likely from the reaction in the dilute phase. And finally, to test whether polyanion identity had any major effect on template-directed polymerization, we performed template-directed polymerization under coacervation conditions using oligoaspartic acid (D_10_) with different polycations. Oligoaspartic acid was chosen because Asp is considered one of the early amino acids^33^. Product yields after 1.5 h were similar for reactions performed in buffer, and in PDAC/rA_11_, PDAC/D_10_, and K_10_/D_10_ coacervates (Supplementary Figure 4c, 27–22%). However, the product yield was significantly reduced for R_10_/D_10_ coacervates (7.5%). These data suggest that oligopeptides can also serve the role of polyanion to form coacervates while supporting template-directed polymerization.

To better understand the differences between the specific coacervates, we performed fluorescence recovery after photobleaching (FRAP) experiments, which can report on physical properties of molecules inside biogenic and abiogenic membraneless compartments^18,19,34^. FRAP of a Cy3-labeled RNA primer (Fig. 2c) revealed significantly faster fluorescence recovery in PDAC-53/rA_11_ as compared to R_10_/rA_11_ coacervates. Half-times for fluorescence recovery (*t*_1/2_) were 27 ± 3 s for PDAC/rA_11_ coacervates and 101 ± 6 s for R_10_/rA_11_ coacervates. Structural analyses have shown that arginine dominates as the most frequent amino acid at the binding interface of RNA aptamers and their protein targets^35^, presumably due to its ability to interact with nucleic acids via multiple mechanisms. Strong arginine–RNA interactions could also explain slower RNA diffusion within this condensed phase and poor RNA polymerization (see above). FRAP experiments where coacervates contained Cy3-labeled RNA primer complexed with the template showed a similar trend, with the half-times for fluorescence recovery (*t*_1/2_) of 49 ± 5 s and 250 ± 64 s for PDAC-53/rA_11_ and R_10_/rA_11_ coacervates, respectively (Supplementary Figure 5a). Overall, the apparent diffusion coefficients were fourfold to fivefold higher for PDAC-53/rA_11_ compared to R_10_/rA_11_ coacervates, regardless of whether the RNA primer was single-stranded or part of the duplex with the template (Supplementary Figure 5b).

### Polyamines rescue polymerization at sub-optimal magnesium levels

**Fig. 3:**
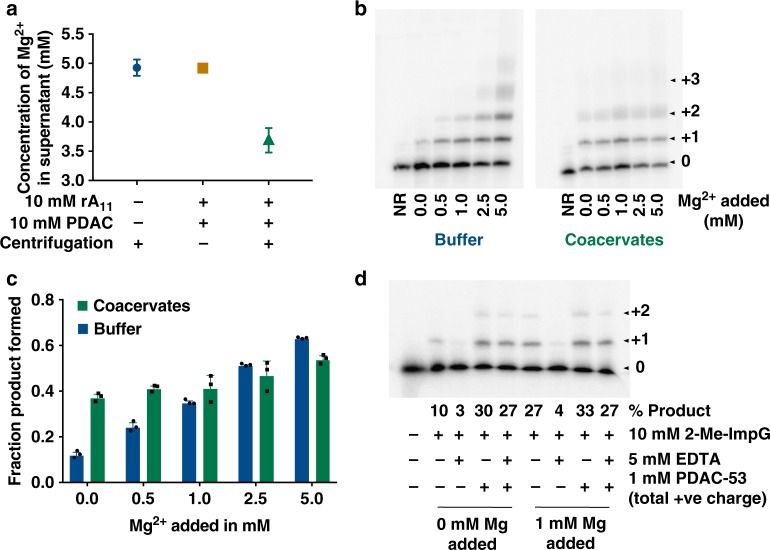
Template-directed polymerization at sub-optimal Mg concentrations. **a** Mg ^2+^ levels were measured by atomic absorption spectroscopy. Solutions with 5 mM MgCl_2_ were formed in 25 mM Tris–HCl pH 8.0 with indicated anions and cations, followed by centrifugation at 14,000×*g* for 2 min. A portion of the supernatant phase was removed and diluted in water prior to measurement. Error bars represent the range of values from two experiments. **b** Yields of template-directed polymerization at different amounts of added Mg in presence and absence of PDAC-53/rA_11_ coacervates. **c** Quantifications from **b**. Error bars represent S.E.M. from three independent experiments. **d** Template-directed polymerization reactions were assembled as previously described in the presence or absence of indicated molecules and ions. Uncropped gel images are shown in Supplementary Figure 15a, b

We previously reported high Mg^2+^ concentration inside PAH/ATP coacervates^17^. To test whether PDAC/rA_11_ coacervates also concentrate Mg^2+^, we measured Mg^2+^ levels inside the condensed phase using Atomic Absorption Spectroscopy (AAS). We estimated the volumes of PDAC/rA_11_ coacervates by centrifuging the bulk coacervate solution and comparing to standards, where 20 µL of bulk solution gave about 1 µL coacervate phase (Supplementary Figure 6a). To calculate the Mg^2+^ levels, coacervate solutions with 5 mM total Mg^2+^ were prepared. Samples were centrifuged and the Mg^2+^ remaining in the supernatant was measured (Supplementary Figure 6b). About ~27% of the total Mg^2+^ is localized to the coacervate phase for PDAC/rA_11_ system. Based on estimated 1 µL coacervate volume, this corresponds to 26 ± 3 mM Mg^2+^ (Fig. 3a).

To determine whether coacervation by PDAC/rA_11_ would facilitate template-directed polymerization of RNA at sub-optimal total magnesium concentration, we performed the reactions at different amounts of Mg^2+^ (Fig. 3b). Above 1 mM Mg^2+^, no gain in product yield under coacervation compared to buffer conditions was evident. However, in the presence of only 0.5 mM Mg^2+^, an increase in product yield in coacervate solutions over buffer was observed. It should be noted that while we estimate roughly 26 mM Mg^2+^ in the coacervates, the yields of polymerization in 5.0 mM added Mg^2+^ are not commensurate with a fivefold increase in Mg^2+^, suggesting that Mg^2+^ concentration alone is not sufficient to enhance template-directed polymerization in coacervates. To our surprise, we saw significant product formation even when magnesium was completely omitted from the buffer (Fig. 3b, c). These data indicate that the positive charge in PDAC may actively participate in chemistry in the absence of metal ions.

To further investigate this possibility, we performed template-directed reactions in 0 and 1 mM added MgCl_2_ in the absence of rA_11_, i.e., no polyanion for coacervation (Fig. 3d). In this experiment, 5 mM EDTA was added to chelate background divalents that may contribute to catalysis. Addition of EDTA essentially halted the slow reaction in the absence of added Mg^2+^ (Fig. 3d, lane 3), suggesting that EDTA served to sequester trace divalent ions. Surprisingly, in the presence of 5 mM EDTA and no added Mg^2+^, the yield of template-directed polymerization was enhanced eightfold by PDAC-53 (Fig. 3d, lane 5 and Supplementary Figure 7a). Addition of 5 mM EDTA stopped the polymerization reaction in 1 mM Mg^2+^ (27% vs 4% yield), which was restored by PDAC-53 to the same extent as in no added Mg^2+^, as expected (4% vs 27%) (Fig. 3d, lanes 7 and 9, and Supplementary Figure 7a). Moreover, 1 mM positive charge in PDAC-53 activates the reaction to the same extent as 1 mM Mg^2+^ (Fig. 3d, lanes 8 and 9). Addition of 1 mM EDTA to reactions containing 1 mM MgCl_2_ inhibited the polymerization, while it did not significantly affect reactions containing 5 mM MgCl_2_ (Supplementary Figure 7b, lanes 2 and 3 vs 6 and 7), indicating that inhibition by EDTA is indeed due to Mg^2+^ sequestration and that EDTA itself does not inhibit the reaction. Furthermore, unlike PDAC-53 polymer, tetramethyammonium monomers did not rescue the polymerization in the presence of EDTA (Supplementary Figure 7c). These data suggest that enhancements in polymerization is likely from favorable contributions from the polyamine polymer.

### Active RNA aptamer inside coacervates

**Fig. 4:**
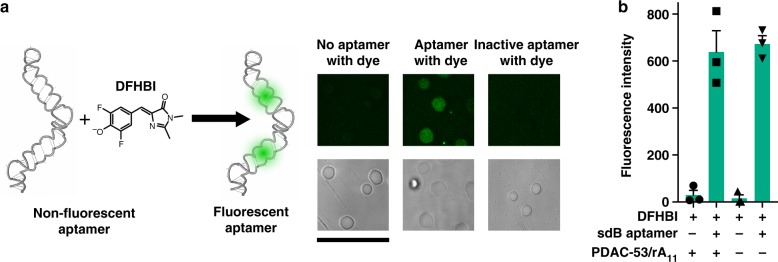
Active RNA aptamer folding inside PDAC/rA_11_ coacervates. **a** (Left) Model of stabilized dimeric broccoli (sdB) RNA drawn in NUPACK^55,^ and confocal microscopy images (right) of PDAC-53/rA_11_ coacervates containing sdB RNA complexed with DFHBI dye. No fluorescence is seen when coacervates contain only DFHBI dye or an inactive mutant of the aptamer. Coacervates contained 100 nM sdB RNA and 10 µM DFHBI in 25 mM Tris pH 8.0, 5 mM MgCl_2_ and 5 mM KCl. 488 nm laser was used for excitation and emission window was 500–550 nm. Scale bar is 20 µm. **b** Bulk fluorescence measurement of sdB RNA aptamer in the presence and absence of PDAC-53/rA_11_ coacervates. Error bars represent S.E.M. (*n* = 3) from three independent experiments

Small molecule-binding aptamer function is highly relevant to origin of life, as metabolic ribozymes likely needed to form binding pockets to bind small metabolites. The broccoli aptamer, which is similar to Spinach aptamer^36^, uses a precise 3D RNA structure to bind to the dye 3,5-difluoro-4-hydroxybenzylidene imidazolinone (DFHBI) and activate its fluorescence^37^. In vitro transcription reactions of Spinach aptamer have been performed within spermidine/polyU complex coacervates^38^. We sought to understand whether, and how well, PDAC-53/rA11 coacervates could support RNA aptamer activity. When DFHBI was added alone to PDAC-53/rA_11_ coacervates, no fluorescence was observed within the droplets, showing that coacervates alone do not induce fluorescence from DFHBI (Fig. 4a). Next, cotranscriptional fluorescence was measured for stabilized dimeric broccoli^39^ (sdB) aptamer and its inactive mutant to verify aptamer-induced fluorescence (Supplementary Figure 8a). Purified sdB aptamer was complexed with the dye and added to PDAC-53/rA_11_ coacervate-containing mixture. Fluorescent coacervate droplets indicated that the sdB aptamer remained natively folded inside coacervates (Fig. 4a). Importantly, no fluorescence was observed in coacervates with the G63C/G87C binding site mutant (equivalent G in each aptamer domain) (Fig. 4a and Supplementary Figure 8a)^37^. Additionally, the droplets were fluorescent when the RNA was allowed to diffuse into coacervates already containing ligand or when the ligand was allowed to diffuse into coacervates already containing the RNA (Supplementary Figure 8b). These data indicate that appropriate small molecules and RNAs can be readily taken up by the coacervates and remain natively folded. Similar fluorescence intensities were measured when sdB aptamer–DFHBI complex was in buffer as compared to PDAC-53/rA11 coacervates, while the fluorescence remained close to background in the absence of the RNA aptamer in buffer and in coacervates (Fig. 4b). These data suggest that the fraction of RNA aptamer that is folded to a functional form is similar in coacervates as in buffer.

### Coacervates enhance ribozyme activity

**Fig. 5:**
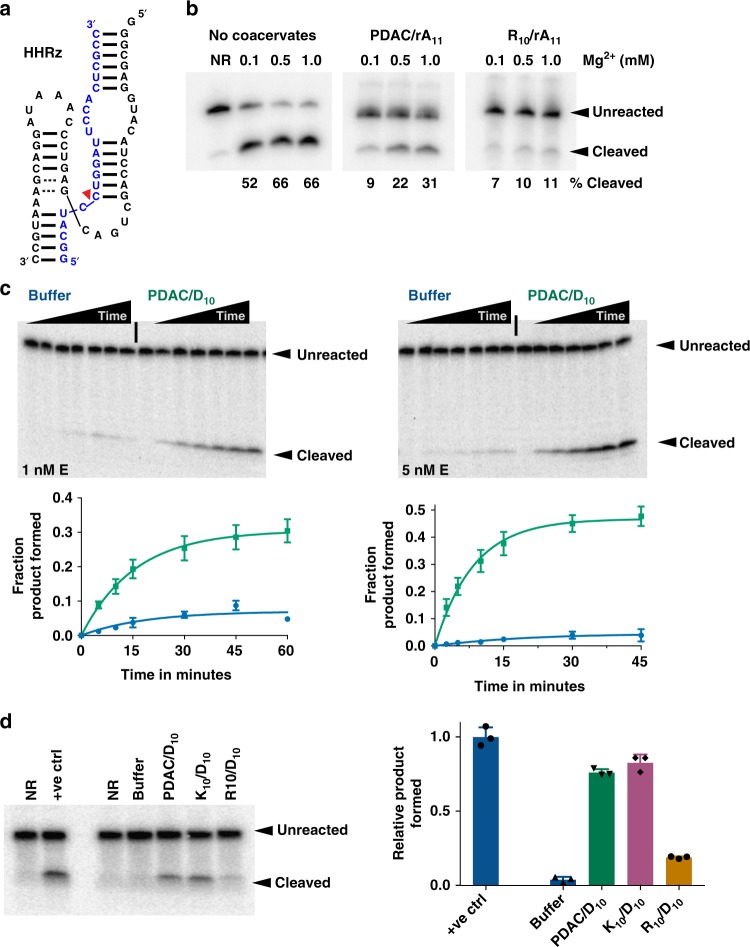
Enhanced ribozyme activity in coacervates. **a** Structure of the hammerhead ribozyme–substrate complex. The cleavage site is indicated with red arrow. **b** Gel images showing ribozyme cleavage at different Mg^2+^ concentrations in the coacervate phase after 1 h. Dilute and coacervate phases were separated immediately after the reaction initiation. Reactions contained 25 mM Tris·HCl pH 8.0, 5 mM NaCl and indicated Mg^2+^. **c** Representative gel images showing ribozyme cleavage in buffer or in PDAC-53/D_10_ coacervates at 1 nM and 5 nM ribozyme concentration; data were quantified from the gels above and fit to Eq. (1). Reactions in buffer (blue trace) and PDAC/D_10_ coacervates (green trace) were performed at room temperature in 25 mM Tris–HCl pH 8.0, 1 mM MgCl_2_, and 2.5 mM KCl. For 1 and 5 nM enzyme, observed rate constants are 0.05 ± 0.02 and 0.05 ± 0.02 min^−1^ for reactions in buffer and 0.06 ± 0.01 and 0.12 ± 0.01 for reactions in coacervates. **d** Representative gel image showing ribozyme cleavage after 1 h of reaction in 25 mM Tris–HCl pH 7.5, 1 mM MgCl_2_, and 2.5 mM KCl. NR contained no ribozyme, +ve ctrl contained 250 nM ribozyme, and all other lanes contained 2.5 nM enzyme. Coacervates contained 15 mM total positive charge from various polycations and 15 mM total negative charge from D_10._ Product yields were normalized to “+ve ctrl”. All error bars represent S.E.M. (*n* = 3) from three independent experiments. Uncropped gel images are shown in Supplementary Figure 16a, b

After studying RNA polymerization and RNA aptamer function, we explored whether PDAC-53/rA_11_ coacervates provide suitable reaction conditions for ribozyme catalysis. We evaluated full-length hammerhead ribozyme (HHRz) cleavage reactions (Fig. 5a) in coacervate phases (separated from supernatant phases) of either PDAC-53/rA_11_ or R_10_/rA_11_ coacervates. The fraction cleaved was relatively smaller for both PDAC/rA_11_ and R_10_/rA_11_ coacervates compared to buffer after 1 h. At 1 mM Mg^2+^, yields were 66%, 31%, and 11% for buffer, PDAC/rA_11_, and R_10_/rA_11_ coacervates, respectively. The yields were considerably higher (~3-fold) for PDAC-53/rA_11_ coacervates compared to R_10_. As expected, almost no signal was observed in the supernatant phase after partitioning of coacervate phase (Supplementary Figure 9a). These data suggest that PDAC-53/rA_11_ coacervates support not only template-directed RNA polymerization, but also catalytic RNA functions.

Next, we asked whether coacervates could actually enhance ribozyme activity. Enhanced activity of the hammerhead ribozyme has been previously reported in aqueous two-phase systems (ATPS) of PEG and dextran by our labs^28^. We sought to understand whether concentration of biomolecules by coacervation can also lead to enhancements in ribozyme reactions at low enzyme concentrations. To explore this possibility, we measured the apparent dissociation constant (*K*_D_) between the ribozyme and the substrate to be 220 ± 10 nM at 1 mM MgCl_2_ and 28 ± 3 nM at 25 mM MgCl_2_ in the background of 2.5 mM KCl (Supplementary Figure 9b). We then estimated the ribozyme concentration inside PDAC-53 and oligoaspartic acid (D_10_) coacervates by using hammerhead ribozyme with a fluorescent tag, and comparing the fluorescence inside coacervates to standards. PDAC-53/D_10_ coacervates indeed concentrated the ribozyme, where the concentration of enzyme strand inside the coacervate was measured to be 45 ± 3, 110 ± 5, and 140 ± 9 µM when only 10, 25, and 50 nM of fluorescently labeled ribozyme was added to the bulk coacervates, respectively (Supplementary Figure 10).

To investigate whether this ribozyme accumulation in coacervates could enhance catalysis, we performed hammerhead cleavage reactions in the presence of PDAC-53/D_10_ coacervates. When concentrations of the enzyme strand were reduced to 1 nM and 5 nM in 1 mM Mg^2+^, i.e., below bulk *K*_D_ to remain under *k*_cat_/*K*_M_ conditions, ribozyme cleavage was significantly reduced with only maximal cleavage of 4–6% (Fig. 5c). Surprisingly, even at these concentrations, 33–45% cleaved product was observed when the reaction was performed in the presence of PDAC/D_10_ coacervates. In other words, reactions performed in buffer alone gave 5–10× less product at any given point in time. Importantly, reactions performed in the presence of either PDAC-53 or D_10_ alone did not enhance the ribozyme activity (Supplementary Figure 11), demonstrating that the mechanism of enhancement is due to coacervation. Finally, to test whether the enhancement of ribozyme reaction is polycation-specific, we performed the reaction in the presence of coacervates containing PDAC, K_10_, or R_10_ as polycations and D_10_ as the polyanion. Under low enzyme concentration, both PDAC- and K_10_-containing coacervates showed greatly enhanced product yield compared to buffer (Fig. 5d) while the product yields in the presence of R_10_-containing coacervates were relatively low. Taken together, these data indicate that complex coacervates can not only support RNA catalysis as previously described^30^, but also provide a microenvironment that enhances RNA catalysis when RNA is scarce by concentrating RNA molecules.

### Activation of multi-domain split ribozyme by coacervates

**Fig. 6:**
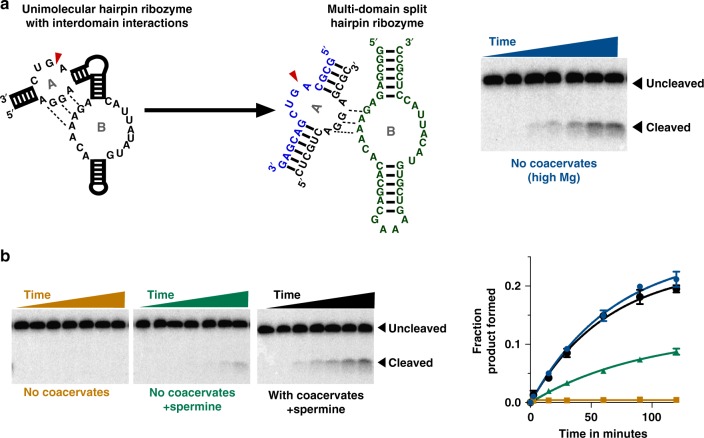
Concentration of cofactors in coacervates enhances of hairpin ribozyme catalysis. **a** Consensus secondary structure of the unimolecular hairpin ribozyme with conserved nucleotides (left), the site for cleavage is indicated with red arrowhead. Multi-domain split hairpin ribozyme where loop A consists of the substrate (blue) and the substrate-binding strand (black) and loop B domains separated (green). Gel image showing cleavage of the substrate is shown. Reaction were performed in buffer containing 25 mM Tris–HCl pH 7.5, 25 mM MgCl_2_ and 25 mM KCl. Reactions contained 1 nM substrate, 1 µM substrate-binding strand, and 1 µM of the loop B domain. **b** Substrate cleavage of loop A domain by the ribozyme at sub-optimal conditions. All reactions were performed in 25 mM Tris·HCl pH 7.5, 2.5 mM MgCl_2_, and 2.5 mM KCl with additional components as indicated. Reactions contained 1 nM substrate, 50 nM substrate-binding strand, and 25 nM of the loop B domain. Coacervates were formed by PDAC-53 and spermine at 5 mM total positive charge from each and 10 mM total negative charge from polyaspartic acid (D_30_). Time points were taken at 0, 2.5, 15, 30, 60, 90, and 120 min. Plots showing fraction product cleaved versus time is shown in right. Fractions product formed were fit to Eq. (1). Colors in the plot correspond to labels of gel images. All error bars represent S.E.M. (*n* = 3) from three independent experiments. Uncropped gel images are shown in Supplementary Figure 17a

Another potential mechanism for reaction activation by coacervates is their ability to concentrate small molecules, which can serve as cofactors in catalysis^17,18^. To evaluate this possibility, we investigated the multi-domain split hairpin ribozyme^40^ reaction which is stimulated by spermine at low magnesium concentrations^41^ (Fig. 6a). In the multi-domain split Hairpin ribozyme, the complex of substrate (blue) and the substrate-binding strand (black) form the “loop A” of the ribozyme system. The “loop B” is formed by a separate enzyme strand (green). We first validated previously reported^40^ cleavage activity of the multi-domain split hairpin ribozyme in 25 mM MgCl_2_ and 25 mM KCl at pH 7.5, and under saturating concentrations of the enzyme and the substrate-binding strands in the absence of coacervates. Under these conditions, cleavage of substrate was readily observed (Fig. 6a, gel image and 6b, blue trace). When the loop B and the substrate-binding strands were reduced to 25 nM and 50 nM, respectively, in buffer containing 2.5 mM MgCl_2_ and 2.5 mM KCl, no detectable product was observed (Fig. 6b, left image and orange trace). A slight increase in product yield was observed when spermine (5 mM total positive charge) was added to the reaction, in the absence of coacervates (Fig. 6b, middle image and green trace), but the overall yields were significantly lower compared to saturating concentrations of loop A and loop B. We then performed the reaction in a hybrid coacervate system of PDAC-53 and spermine as polycations with polyaspartic acid, D_30_, as the polyanion. Surprisingly, under these conditions, the ribozyme cleavage was significantly enhanced compared to reactions in low Mg^2+^ and K^+^ without coacervates (Fig. 6b, right image and black trace). Importantly, when only PDAC-53 and D_30_ are present no enhancement in ribozyme activity is observed (Supplementary Figure 12b) despite the presence of coacervates. On a similar note, when spermine and D_30_ are present in the reaction, no enhancement is observed, and under these conditions no coacervates are present (Supplementary Figure 12c). Taken together, these data indicate that complex coacervates can also enhance ribozyme catalysis by encapsulating cofactors that stimulate the reactions.

### Coacervate-mediated enhancement of a DNAzyme

**Fig. 7:**
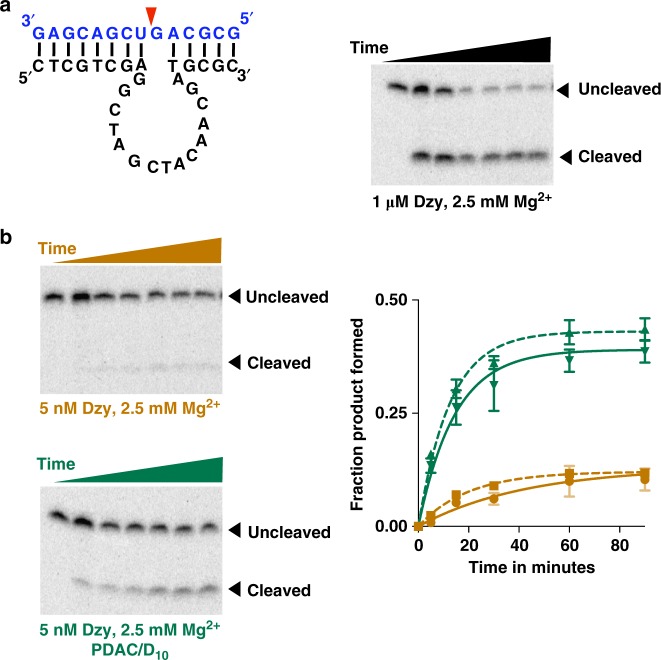
Coacervate-mediated stimulation of a DNAzyme. **a** Structure of the 10–23 DNAzyme. The enzyme strand is shown in black and the substrate is shown in blue. The red arrow indicates the cleavage site. Gel image shows efficient cleavage of the substrate (0.25 pM) by the enzyme (1 µM) in 25 mM Tris–HCl pH 8.0 containing 2.5 mM MgCl_2_ and 2.5 mM KCl. Time points were taken at 0, 2.5, 5, 15, 30, 60, and 90 min. **b** Reactions contained 5 nM of the enzyme strand and 0.25 pM substrate strand in 25 mM Tris–HCl pH 8.0 containing 2.5 mM MgCl_2_ and 2.5 mM KCl. Coacervates were formed by adding PDAC-53 and D_10_ at 10 mM total charge from each. Time points were taken at 0, 5, 15, 30, 60, 90, and 120 min. Fraction product formed were calculated from gels shown in **b** and Supplementary Figure 13 and data were fit to Eq. (1). Green solid and dashed lines indicate experiments performed in 2.5 mM Mg^2+^ or 5 mM Mg^2+^, respectively. All error bars represent S.E.M. (*n* = 3) from three independent experiments. Uncropped gel images are shown in Supplementary Figure 17b

As coacervate-dependent enhancement is primarily due to concentration effect, we sought to understand whether it can be applied to other catalytic systems besides RNA enzymes. The 10–23 deoxyribonucleic acid enzyme (DNAzyme) was selected to cleave RNA molecules by in vitro evolution^42^. At 2.5 mM MgCl_2_ and saturating concentrations of the DNAzyme, we observed efficient cleavage of the substrate, with about 65% yield after 1.5 h (Fig. 7a). However, when the enzyme concentration was reduced to 5 nM, the cleavage reaction of the substrate was significantly reduced (10% yield after 2 h) (Fig. 7b, top gel and solid orange trace). In the presence of coacervates, the reaction was partially restored, with fourfold improvement in product yield compared to reactions in buffer alone after 2 h (~40% vs ~10%) (Fig. 7b). Similar coacervation-induced stimulation was observed when the reactions were performed at 5 mM Mg^2+^ (Fig. 7b, dashed traces and Supplementary Figure 13a). Identical electrophoretic mobility shifts of the cleaved products from different reaction conditions support the same cleavage site for all the conditions (Supplementary Figure 13b). Taken together, these data indicate that coacervate-mediated enhancements under sub-optimal conditions is not limited to RNA enzymes.

## Discussion

In this study, we have combined model prebiotic compartmentalization systems of coacervates and essential RNA functions of template-directed RNA polymerization, RNA aptamer–ligand interactions, and ribozyme catalysis. Inhibition of polymerization by PAH-260, R_10_, and K_100_ in the absence of counterions suggests that the highly charged polyamine alone is capable of interfering with template-directed RNA polymerization. It is likely that the extensive charge–charge interactions and H-bonding disrupt the primer–template complex and/or disorient the 3′ OH such that the 3′ O of primer and the P-5′ O bonds are not in line for the nucleophilic attack. Oligoarginine significantly inhibited the reaction whereas oligolysine with the same number of residues did not, although the longer oligolysine (K_100_) did. Arginine can interact extensively with nucleic acids, especially guanine nucleotides in bidentate interactions with O-6 and N-7^35^, possibly interfering with the base-pairing within the primer–template complex and also potentially with the 2-Me-ImpG monomer. The quaternary amine PDAC lacks H-bonding capabilities and has a buried positive charge and therefore may not actively interact with nucleic acids in an inhibitory manner. Although the prebiotic origin of PDAC is not yet known, pyrrolidine, which resembles the unmethylated monomer of PDAC, has been detected in meteorite samples^43^. While PDAC/RNA coacervates outperformed others, it should be emphasized that templated-polymerization did occur in K_10_/RNA coacervates, albeit at slightly reduced levels. Furthermore, when coacervates coexisted with the dilute phase, templated-polymerization in K_10_/RNA and PDAC/RNA were identical to buffer conditions while encapsulating over 65% of the RNA in the coacervate phase. These observations coupled with the recent report of hammerhead catalysis in pLys/CM-Dex coacervates^30^ suggest that RNA chemistries can occur in a wide range of coacervate compositions and that the chemistry of a particular coacervate will impact its effectiveness as a reaction compartment.

One of the intriguing observations was that at reduced or no Mg^2+^, PDAC-53/rA_11_ coacervates and PDAC-53 alone stimulated template-directed polymerization compared to buffer. Since PDAC does not have proton donating or accepting capabilities to act as general acid or general base, the enhancement in reaction could be from charge stabilization of the transition state provided by the positively charged nitrogens of PDAC. These data suggest that coacervate systems can actively participate in the chemistry rather than simply acting as a “compartment” for reactions. To the best of our knowledge, polyamine-dependent enhancement of template-directed polymerization has not been reported; however, enhancement of ribozyme cleavage from spermidine at limiting^44^ and in the absence^41^ of magnesium has been observed. Typically, molar amounts of monovalent are required to substitute for magnesium in ribozyme reactions^45,46^. Coacervates may provide the unique ionic conditions required for activity by concentrating ions. We estimated roughly a 5-fold increase in Mg^2+^ inside PDAC/rA_11_ coacervates, and Mg^2+^ bound weakly to the polyanions may still be available for template-directed polymerization and other related chemistries^47^.

Differential RNA diffusions in PDAC and R_10_ coacervates as observed in FRAP experiments indicate that specific coacervates may provide unique environments and have varying degree of RNA–coacervate interactions. Apparent diffusion coefficients reported here for PDAC-53/rA_11_ and R_10_/rA_11_ are about 400-fold lower compared to spermine/polyU RNA systems where different 15-mer RNAs were used as fluorescent probes^18^. This is likely due to differences in coacervate composition; for example, about 40-fold difference in fluorescence recovery time-constant from FRAP experiments for the same RNA probe in pLys/ATP and pLys/CM-Dextran coacervates has been reported^30^.

An important consideration for a model protocell during the RNA world is that it should be able to maintain activities of compartmentalized aptamers or ribozymes within the protocells. The activity of the fluorescent RNA aptamer inside the coacervate droplets unambiguously shows that PDAC/rA_11_ coacervates support folded RNA secondary and tertiary structures, which is required for many RNA functions. We have shown herein that PDAC/rA_11_ coacervates retain activity of hammerhead ribozyme. Reduced ribozyme activity has recently been reported for the hammerhead cleavage in coacervates made with polylysine and carboxylated dextran^30^ at saturating enzyme concentrations. Under those conditions, about 13-fold to 60-fold reductions in observed rate constants were reported depending on whether the reactions were carried out in microdroplets or in bulk coacervates. Interestingly, in our study, at low ribozyme concentrations, we observe enhancements in ribozyme cleavage yields in the presence of coacervates at all times points. Enhancement of the hammerhead ribozyme was also observed for K_10_/D_10_ coacervates. On a similar note, stimulation of the hairpin ribozyme by spermine-containing coacervates and enhancement of the DNAzyme cleavage by PDAC/D_10_ coacervates suggest that coacervate-dependent activation may be useful for reactions catalyzed by diverse biopolymers and that coacervate-dependent activation is not limited to PDAC-containing coacervates. Taken together, our study suggests that coacervates can potentiate functions of RNA and other biopolymers under sub-optimal enzyme and ion conditions by concentrating biomolecules and solutes, which would have been critical in the early RNA world where concentrations of functional RNAs may have been scarce.

It has been previously demonstrated that protein kinases can phosphorylate polyions such as cationic peptides or ADP to trigger dissolution of complex coacervates, which can be reversed by phosphatase enzymes^48,49^. In an “RNA World”, a ribozyme with kinase^3,50^ or phosphatase activity could have performed similar roles by modulating charge densities on polymers through covalent modification^4^. Transient coacervation of metabolic enzymes and their substrates could have provided micro-reactor compartments for specific reactions that may otherwise be inaccessible in dilute solutions.

## Methods

### Reagents and synthesis

Poly(allyl)amine hydrochloride (PAH) 15 kDa, protamine sulfate, and polyacrylic acid (1.8 kDa) were purchased from Sigma (St. Louis). Polydiallyldimethylammonium chloride (PDAC) 8.5 kDa was purchased from Polysciences (Warrington, PA). Oligolysine, oligoarginine, and oligoaspartic acid were purchased from Alamanda polymers (Huntsville, AL) as chloride or sodium salts. Charge density calculated by assuming that all the protonable nitrogen atoms in the polymer are protonated at the reaction pH. pKas for monomer ionizable groups of PAH, R_10_, and K_10_ are 8.8^51^, 12.48^52^, and 10.53^52^, respectively. RNA primer and templates were purchased from IDT (Coralville, IA). Oligoadenosine (rA11) was purchased from Trilink Biotechnologies (San Diego, CA). Guanosine monophosphate was purchased from MP Biomedicals. 2-Me-ImpG was synthesized as previously described^53^, with some modifications. In brief, 100 mg of GMP-free acid and 240 mg of 2-methyl imidazole were added to 20 ml of DMSO and stirred at 50 °C. To the solution of GMP, 0.75 g of triphenylphosphine, 0.85 g of aldrithiol-2, and 1.08 mL of triethylamine were added. Reactions were allowed to proceed for 1 h at room temperature. Then, the mixture was added dropwise to 100 mL solution of 1:1 acetone, diethyl ether containing 1.1 g sodium perchlorate to precipitate 2-Me-ImpG. Solution was then centrifuged at 5000 × *g* for 5 min and the resulting pellet was washed 4× with ~20 mL 1:1 acetone:diethyl ether solution and then twice with ~20 mL diethyl ether. 2-Me-ImpG was obtained as dried powder by vacuum centrifugation.

### Template-directed polymerization

Template-directed reactions were assembled by first adding water and buffer (1× = 25 mM Tris–HCl pH 8.0, 5 mM MgCl_2_). For reactions not containing any coacervates, cationic polymers were then added to indicated concentrations, 2-Me-ImpG was added to a final concentration of 10 mM, followed by addition of RNA primer–template complex (1 µM template and < 50 nM 5ʹ labeled primer). For reactions done under coacervation conditions, the anionic component of the coacervate system was added following the addition of water and buffer. The total negative charge from the rA_11_ polyanion was held at 7.5 mM. 2-Me-ImpG was then added to a final concentration of 10 mM. Coacervation was induced by addition of different cationic polymers with total positive charge at 10 mM. Reactions were initiated by addition of RNA primer–template complex and incubated at room temperature for indicated times. The total negative charge from the RNA primer–template complex was <20 µM.

Reactions were stopped by adding equal volume of “stopping solution”, which contained 8.3 mM polyacrylic acid 1.8 kDa, 166 mM EDTA and 333 mM NaOH. Finally, one reaction volume of 90% formamide and 50 mM EDTA solution was added after which samples were heated at 85 °C for 1 min and separated by electrophoresis on a 20% denaturing polyacrylamide gel containing 7 M Urea. Gels were dried and exposed to phosphorscreens, which were then scanned by Typhoon FLA 9000 (GE). Fraction of polymerized RNA primer was calculated by using the equation *A*/(*A* + *B*), where *A* is the sum of intensities for polymerized bands, and *B* is the intensity of unreacted primer. For kinetic experiments, data were fit to first order exponential

1 $$f(t) = f_{\mathrm{max}}(1 - {\mathrm e}^{\left( { - k_{\mathrm{obs}} \cdot {t}} \right)})$$

where *f*(*t*) is the fraction product formed at indicated time *t*, *f*_max_ is the plateau, and *k*_obs_ is the observed rate constant. All fits were performed in GraphPad Prism 7. For reactions shown in Fig. 2b, samples were centrifuged immediately for 1 min at 14,000×*g* after addition of the RNA:primer–template complex. The resulting supernatant and the condensed phases were then separated and reactions were allowed to proceed in the respective phases for 5 h at room temperature.

### Hammerhead ribozyme reactions in PDAC/rA_11_ coacervates

Coacervates (10 mM charge balance) were assembled by adding water, buffer (1× = 25 mM Tris–HCl pH 8.0, 5 mM NaCl and 0.1–1 mM MgCl_2_), rA_11_ and then PDAC-53. Ribozyme (10 µM) and substrate (~100 nM) were added in folding buffer that did not contain any magnesium (1× = 25 mM Tris–HCl, 5 mM NaCl). The ribozyme–substrate complex was heated at 85 °C for 3 min and allowed to cool at room temperature for 3 min. Reactions were initiated by adding ribozyme:substrate complex to the Mg^2+^-containing coacervate solutions. Samples were immediately centrifuged at 14,000 × *g* for 1 min. Supernatant and the coacervate phases were separated and allowed to react for 1 h at room temperature. Reactions were stopped for supernatant solution by adding one reaction volume of “stopping solution”, which contained 8.3 mM polyacrylic acid 1.8 kDa, 166 mM EDTA and one reaction volume of formamide loading dye (90% formamide and 50 mM EDTA). For coacervates, water (equal to initial reaction volume) was first added followed by addition of stopping solution. Samples were then heated at 90 °C for 3 min and coacervate phase was disrupted by pipette mixing immediately after heating. Reactions were then separated by electrophoresis on a 15% denaturing polyacrylamide gel containing 8 M Urea.

### Hammerhead ribozyme reactions at low enzyme concentrations

Ribozymes and substrates at 10× of desired concentrations were separately heated at 85 °C for 3 min in water, followed by addition of folding buffer (1× = 25 mM Tris–HCl pH 8.0, 2.5 mM KCl). Ribozyme and substrate were allowed to equilibrate at room temperature for 3 min and kept on ice. To obtain the zero time-point, 10× substrate was added to water. Coacervates (10 mM charge balance) were assembled by adding water, buffer (1× = 25 mM Tris–HCl pH 8.0, 1 mM MgCl_2_, 2.5 mM KCl), oligoaspartic acid D_10_ and PDAC-53. Renatured ribozyme was first added followed by the substrate to initiate the reactions. Reactions were then immediately aliquoted into tubes, and stopped at indicated times. Reactions were then separated by electrophoresis on a 15% denaturing polyacrylamide gel containing 8 M Urea. Plots of fraction cleaved were fit to Eq. (1).

### Estimation of RNA primer concentration inside coacervates

Standards of indicated Cy-3-labeled RNA primer were made in solutions of 25 mM Tris–HCl pH 8.0, 1 mM MgCl_2_. Indicated coacervates were made in 1 mM MgCl_2_ and 25 mM Tris–HCl pH 8.0. A 10× primer–template complex was made by heating the 10 µM Cy3-labeled primer and 15 µM template in water at 85 °C for 3 min followed by addition of 10× buffer (1× = 1 mM MgCl_2_ and 25 mM Tris–HCl pH 8.0). Solution was left at room temperature for 5 min to equilibrate. Indicated coacervates were prepared, and renatured primer–template complex was added such that the primer concentration was 0.5 µM in the coacervate solution. Overall, 8 µL of the solution was placed on a glass slide and mounted on a Leica TCS SP5 laser scanning confocal inverted microscope (LSCM) with a ×63 objective lens. A 543 nm laser was used for excitation and emission spectra were collected from 560 to 600 nm. Leica LAS AF software was used to acquire the images.

### Estimation of coacervate phase volume

Standards of 0.5, 1, 1.5, and 2 µL were prepared by adding indicated amount of Cy3-labeled-RNA primer. These were then centrifuged briefly to ensure that the samples were at the bottom of the tube. A total of 20 µL bulk coacervates of PDAC and rA11 were prepared at 10 mM charge balance in 25 mM Tris–HCl pH 8.0 and 1 mM MgCl_2_. Cy3-labeled-RNA primer was added to a final concentration of 5 µM. Sample was then centrifuged to pellet the condensed phase Samples were placed on UV transilluminator (FOTODYNE Inc.) and irradiated with 312 nm UV while the images were collected. Coacervate phase volume was compared against the known standards by visual inspection.

### Atomic absorption spectroscopy

Standards of 0, 1, 2.5, 5, and 10 µM Mg^2+^ were made using serially diluted commercial 1 M Stock MgCl_2_ (Sigma) in water. Coacervates (20 µL) were prepared in solutions containing 25 mM Tris·HCl and 5 mM MgCl_2_ at 10 mM charge balance. To measure depleted Mg^2+^, samples were centrifuged for 14,000 × *g* for 2 min, and 10 µL of the supernatant was added to 9.99 mL of water. This was also done for samples that did not undergo centrifugation. Samples were then analyzed using a Shimadzu Flame Atomic Absorption Spectrometer 7000. The uncertainty in coacervate volume was not accounted for in estimation of magnesium concentration.

### Ribozyme concentration inside coacervates

Standards of indicated fluorescein were made in solutions of 1 mM MgCl_2_ and 2.5 mM KCl. Hammerhead ribozyme was labeled on the 5′ end with fluorescein as described^54^. In short, in vitro transcribed RNA were treated with calf intestinal phosphatase (NEB) and phosphorylated using polynucleotide kinase (NEB) using ATP-γS. RNAs were then treated with 5-iodoacetamido-fluorescein (Sigma). Labeled RNA was purified using Sephadex G-25 column (GE). Efficiency of labeling and concentration of fluorescently tagged RNA was measured by comparing absorbance at 260 and 490 nm. (*ε*_HHRz_ = 448700 M^−1^ cm^−1^, *ε*_fluorescein_ = 72500 M^−1^ cm^−1^) and calculated to be 35%. Coacervates of PDAC-53 and D10 were made at in 1 mM MgCl_2_ and 2.5 mM KCl and indicated concentrations of fluorescently labeled RNA was added to the droplets. Samples were then imaged as previously described. Overall, 488 nm laser was used for excitation, and the emission window was set from 510 to 550 nm.

### Isothermal binding of the hammerhead ribozyme and substrate

Samples containing 25 mM Tris–HCl pH 8.0, 2.5 mM KCl, 4 pM substrate, and various concentrations of enzyme ranging from 0 to 17.5 μM were incubated at 85 °C for 3 min and then placed at room temperature for a minimum of 3 min. Each sample was then mixed with MgCl_2_ to a final concentration of 1.0 mM or 25 mM and subsequently placed on ice. The RNA was fractionated using native PAGE at 25 °C and imaged via phosphorimager. Fraction of substrate bound at indicated enzyme concentration was determined by quantification of the bands (ImageJ). Data were fit to

2 $${f_{\mathrm b}} = \frac{{B_{{\mathrm{max}}} \cdot X}}{{K_{{\mathrm{d}}} + X}} + C,$$

where *f*_b_ is the fraction bound, *B*_max_ is the maximum bound, *K*_d_ is the dissociation constant, *X* is the concentration of the enzyme and *C* is a constant.

### Fluorescence recovery after photobleaching (FRAP)

After preparing coacervates as described, droplets for FRAP analysis were chosen in the FRAP Wizard interface in the Leica LAS AF software. A region of interest (ROI) of 1 µM diameter was defined for fluorescence measurements during pre-bleach, bleach, and post-bleach sequences. Overall, 5 frames at 0.50 s/frame pre-bleach, 5 frames at 0.19 s/frame during bleach, and 300 frames at 0.5 s/frame post-bleach were acquired. Laser power was set at 100% for 458, 476, 488, 514, 543, and 633 nm lasers for bleach. Fluorescence intensities during the pre-bleach, bleach, and post-bleach sequences were measured in FIJI (Fiji Is Just ImageJ) to generate recovery curves. Recovery data were normalized as described by Jia et al.^19^ by using the equation:

3 $$F( t ) = \frac{{[ {S( t ) - B( t )} ] - [R( o ) - B( o )]}}{{[ {R( t ) - B( t )} ] - [S( o ) - B( o )]}},$$

where *F*(*t*) is the normalized fluorescence intensity of the ROI at the given time. *S*(*t*) is the intensity within the chosen sample ROI for bleaching; *R*(*t*), the intensity within a different droplet that is not bleached; and *B*(*t*), is the intensity of the background ROI.

Data were then fit to the first order exponential

4 $$f( t ) = A(1 - {\mathrm e}^{( - t/\tau )}) + C,$$

where *f*(*t*) is the normalized fluorescence at time *t*, *A* is the amplitude of recovery, and *C* is the *Y*-intercept. Half-lives (*t*_(1/2)_) of fluorescence recovery was calculated by using *t*_(1/2)_ = ln(2)*τ. Apparent diffusion coefficient (*D*_app_) was calculated by the formula *D*_app_ = (0.88ω^2^)/4·*t*_1/2_, where *ω* is the radius of the bleached ROI.

### Fluorescence of stabilized dimeric broccoli (sdB)

The stabilized dimeric broccoli aptamer (GAGGGAGACGGUCGGGUCCAUCUGAGACGGUCGGGUCCAGAUAUUCGUAUCUGUCGAGUAGA**G**UGUGGGCUCAGAUGUCGAGUAGA**G**UGUGGGCUCCCUC) was transcribed in vitro, purified by PAGE and concentrated by ethanol precipitation. The bolded and underlined Gs were mutated to Cs for the inactive mutant. RNA was renatured by heating in water at 85 °C for 3 min, followed by immediately adding the 10× buffer (1× = 25 mM Tris–HCl pH 8.0, 5 mM MgCl_2_, and 5 mM KCl). RNA was left at room temperature for 5 min after which, the DFHBI dye was added, and samples were left on ice until ready to be mixed with coacervate solutions. PDAC and rA11 coacervates were made as previously described. Overall, 1 µL of the folded RNA was added to 14 µL of the coacervate solution, and 10 µL of this sample was used to prepare slides. For samples in which sdB or DFHBI were added separately, coacervates were first formed after adding either DFHBI or folded sdB, then the other component was added. 15% of 453 and 476 nm lasers were used together for excitation, and the emission window was set at 500–550 nm. For bulk measurements, fluorescence of samples in the presence and absence of coacervates was measured at 15 °C in Applied Biosystem StepOne Plus qPCR machine using emission filter for Fluorescein amidite (FAM) (Em. Max = 520 nm).

### Hairpin ribozyme reaction

10× solutions of ribozyme (loop B) was prepared in 25 mM Tris–HCl pH 7.5 1.25 mM MgCl_2_ and 1.25 mM KCl. Ribozyme solution were first heated to 85 °C for 3 min and equilibrated at room temperature for 5–10 min. Similarly, 5× solutions of the substrate·substrate-binding strand complex was also prepared in the same buffer and renatured separately. PDAC-53, Spermine and D_30_ were first assembled in 25 mM Tris pH 7.5 with either 25 mM MgCl_2_ and 25 mM KCl (high Mg) or 2.5 mM MgCl_2_ and 2.5 mM KCl (low Mg). 10× solution of ribozyme was added to indicated final concentrations followed by addition of the 5× substrate–substrate-binding strand complex to initiate the reaction. Reactions were stopped by adding two volumes of the stopping solution which contained 8.3 mM polyacrylic acid 1.8 kDa, 166 mM EDTA, and one volume of 90% formamide loading dye.

### DNAzyme reactions

10× solutions of DNAzyme was prepared in 25 mM Tris–HCl pH 8.0 and 2.5 mM KCl. DNAzyme solutions were first heated to 85 °C for 3 min and equilibrated at room temperature for 5–10 min. Similarly, 10× solutions of the substrate was also prepared in the same buffer and renatured separately. PDAC-53, D_10_ were first assembled in 25 mM Tris pH 8.0 with either 2.5 mM MgCl_2_ and 2.5 mM KCl or 5 mM MgCl_2_ and 5 mM KCl. 10× solution of ribozyme was added to indicated final concentrations followed by addition of the 10× substrate strand to initiate the reaction. Reactions were stopped by adding two volumes of the stopping solution which contained 8.3 mM polyacrylic acid 1.8 kDa, 166 mM EDTA, and one volume of 90% formamide loading dye.

### Reporting Summary

Further information on experimental design is available in the Nature Research Reporting Summary linked to this Article.

## Supplementary Information

Supplementary Information

Reporting Summary

Peer Review File

## Data Availability

The authors declare that data supporting the findings of this study are available within the paper and its Supplementary Information files. All relevant data are available from the corresponding authors upon reasonable request.
