# Supplementary material for: Template-directed RNA polymerization and enhanced ribozyme catalysis inside membraneless compartments formed by coacervates
Source: Nat Commun. 2019 Jan 30;10:490. doi: 10.1038/s41467-019-08353-4 (PMC6353945; doi:10.1038/s41467-019-08353-4)
Supplement: Supplementary file 1 — Supplementary Information [file 41467_2019_8353_MOESM1_ESM.pdf]

Template-directed RNA polymerization and enhanced ribozyme catalysis inside membraneless compartments formed by coacervates

Raghav R. Poudyal[1,2*], Rebecca M. Guth-Metzler[3,4], Andrew J. Veenis[1,2], Erica A. Frankel[1,2,5], Christine D. Keating[1*], Philip C. Bevilacqua[1,2,3*]

[1]Department of Chemistry, [2]Center for RNA Molecular Biology, [3]Department of Biochemistry, Microbiology, and Molecular Biology, The Pennsylvania State University, University Park, Pennsylvania 16802, United States.

[4]Present address

School of Chemistry and Biochemistry, Georgia Institute of Technology, Atlanta, GA, 30332

[5]Present address

The Dow Chemical Company, 400 Arcola Road, Collegeville, PA 19426

*Authors to whom correspondence should be addressed:

rup34@psu.edu

keating@chem.psu.edu

pcb5@psu.edu

**Contents**	**Page**
Supplementary Figure S1-S17	2-18

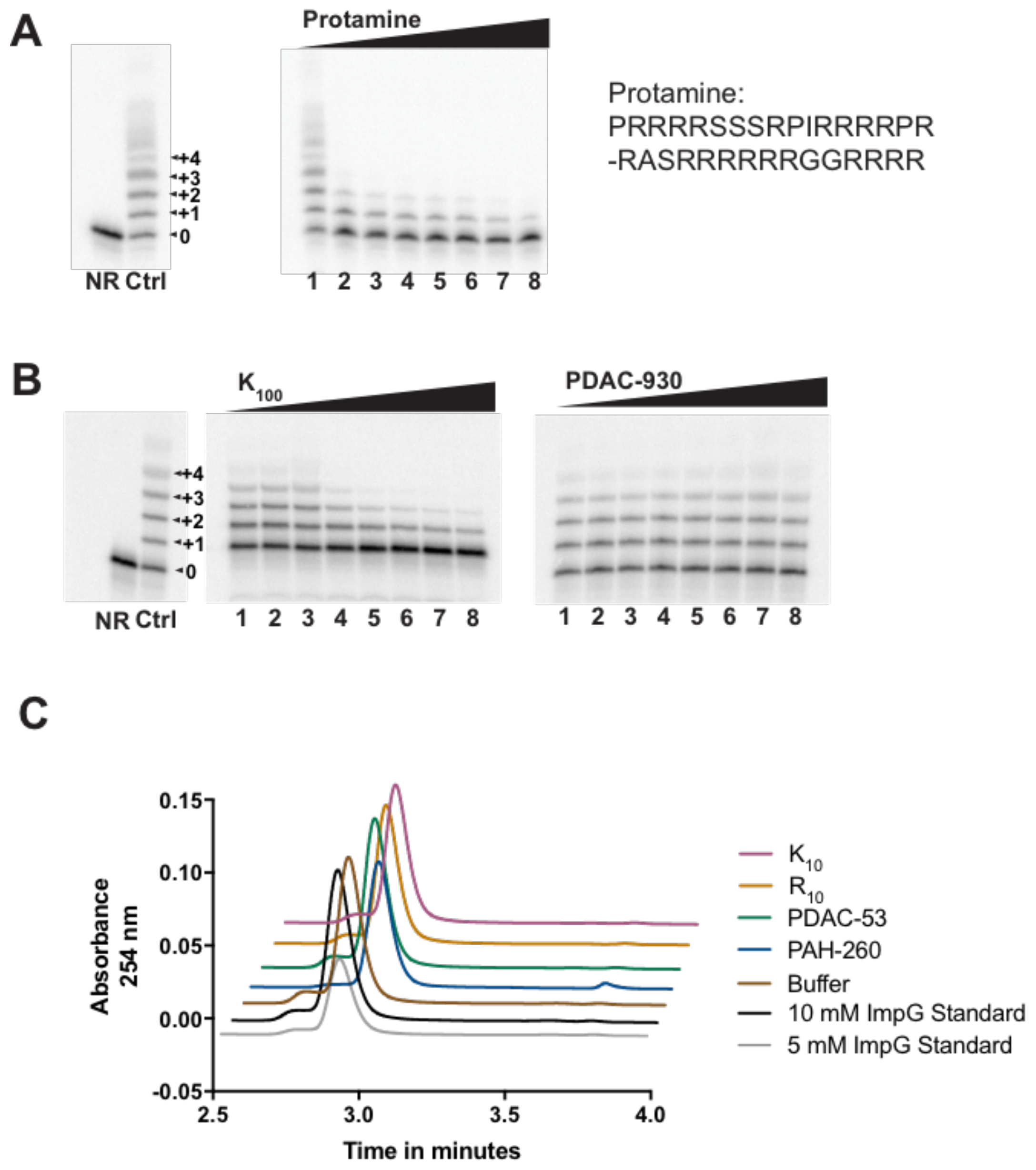

Supplementary Figure 1. Effect of polyamines on non-enzymatic polymerization. (A) and (B) Non-enzymatic polymerization in the presence of varying amounts of (A) protamine and (B) 100mer oligolysine K100 and PDAC-930 (~930 monomers, 150 kDa). Lanes 1-8 contain 0.050, 0.10, 0.50, 1.0, 2.5, 5.0, 7.5 and 10 mM total positive charge. “Ctrl” lane indicates reaction without any cationic polymer. (C) 2-Me-ImpG was incubated with coacervates containing 10 mM total positive charge for 5 h in Tris·HCl pH 8.0 in 5 mM Mg^{2+}. 5 µL of 1/100 dilution of samples were analyzed by reverse-phase HPLC using Waters UHPLC instrument. The following gradient was set: 0 min 100% Buffer A, 10 min 5% Buffer B, 15 min 50% Buffer B, and 20 min 100% Buffer B. Buffer A was100 mM ammonium acetate, 2% acetonitrile and buffer B was 100 mM ammonium acetate and 50% acetonitrile.

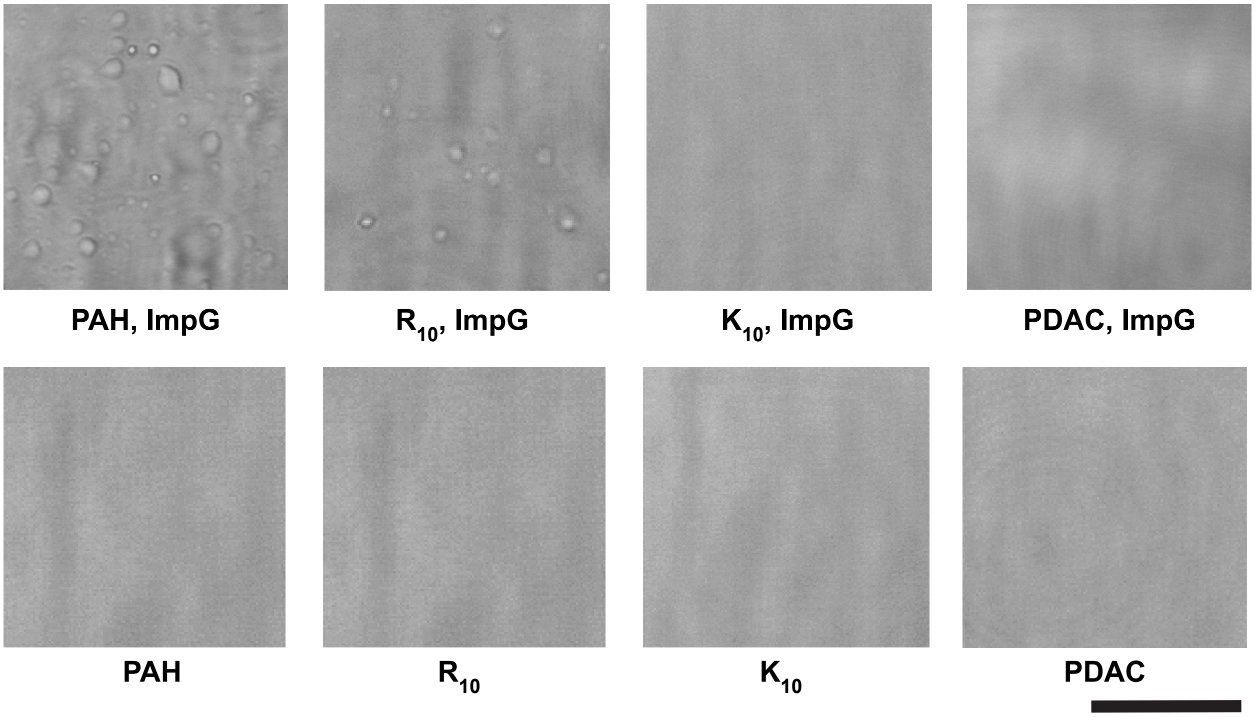

Supplementary Figure 2. Some polyamines can form condensed phases with 2-Me-ImpG. Solutions contained 1 mM total positive charge from polyamines and 10 mM 2-Me-ImpG. All solutions were centrifuged prior to mixing to eliminate any insoluble impurities prior to loading on the coverslips. Transmitted light images were collected on a Leica TCS SP5 microscope. Scale bar is 10 μm

A

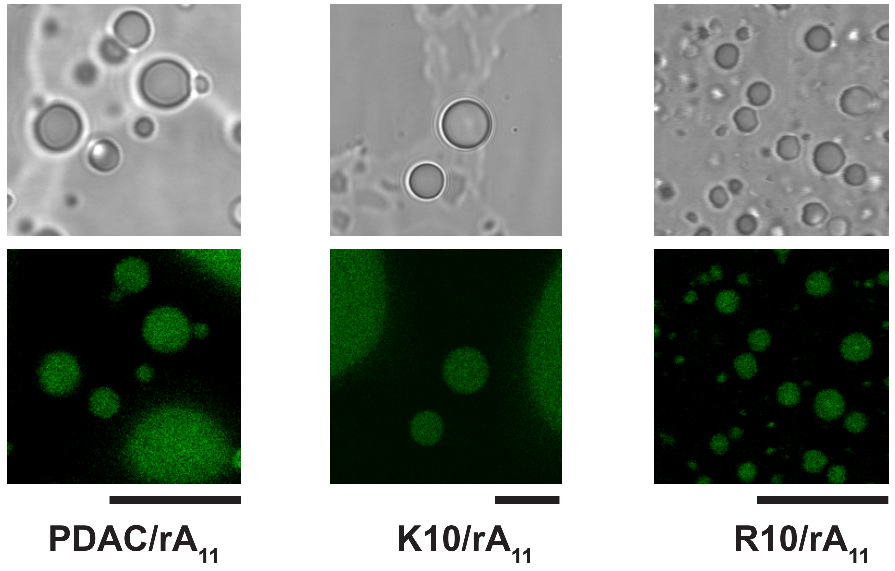

B

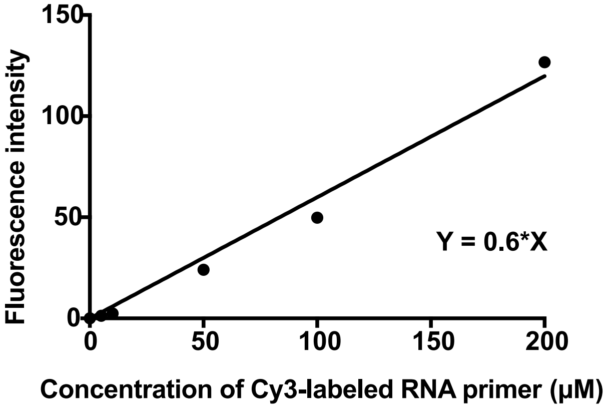

Cy3-labeled RNA primer (μM)		
Cation/Anion	Added	Inside
PDAC-53/rA11	0.5	78±6
K10/rA11	0.5	34±1
R10/rA11	0.5	56±12

Supplementary Figure 3. Increased concentration of primer-template complex inside coacervates. (A) Different coacervates were formed in 25 mM Tris·HCl pH 8.0 and 1 mM $MgCl_2$. Each solution contained 0.5 μM Cy-3 labeled primer and 0.75 μM of the unlabeled template. Scale bar= 10 μm (B) Concentration of Cy3-labeled RNA inside coacervates was estimated by comparing the fluorescence reading with the standards, which were made in 25 mM Tris·HCl (pH 8.0) and 1 mM $MgCl_2$. Errors represent standard error of mean of at least three independent measurements. The R^2 has a value of 0.99.

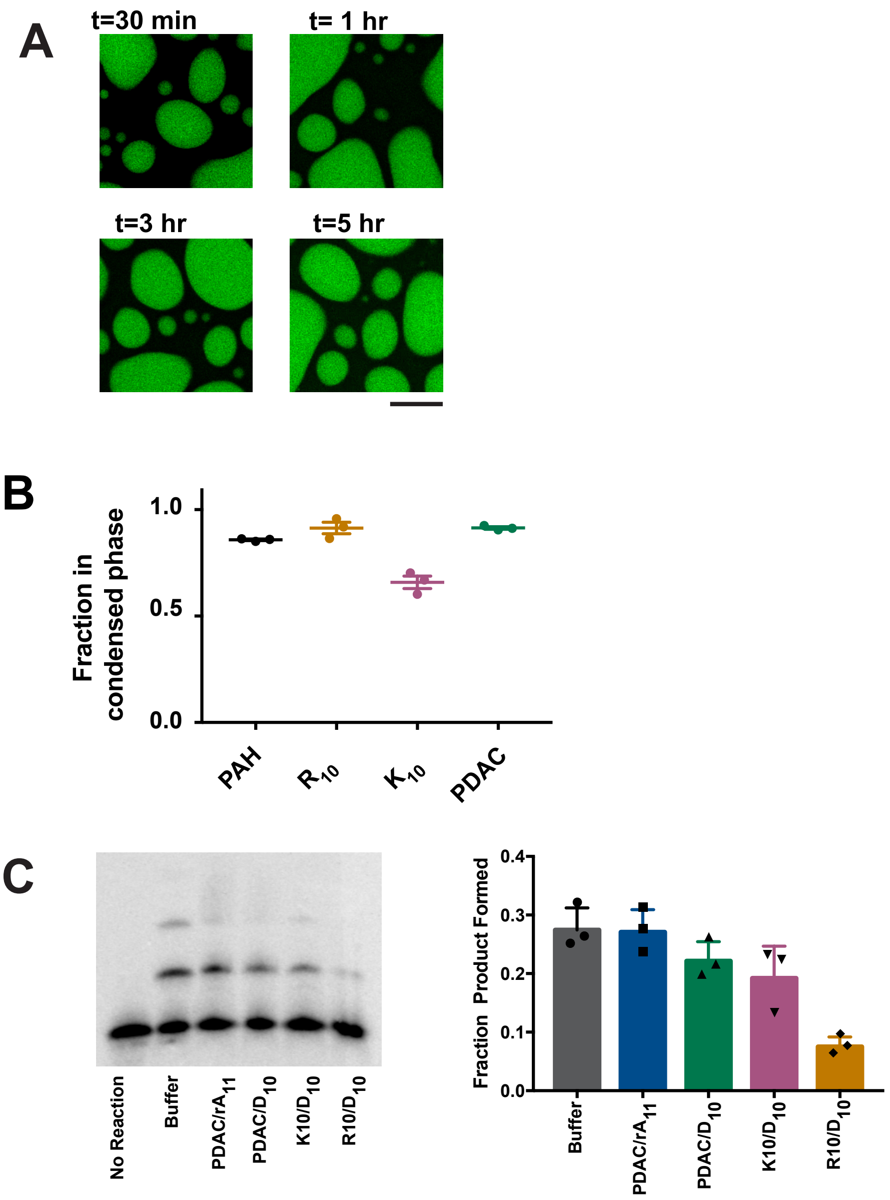

Supplementary Figure 4. PDAC/rA11 coacervates are stable for extended periods and partition RNA. (A) Coacervates were formed at 10 mM charge balanced conditions. 1 µM Cy3 labeled RNA primer was added and images were collected in Leica TCS SP5 microscope at 63 X. Scale bar= 10 µm (B) Fraction RNA in the condensed phase calculated by separating the condensed phase from the supernatant phase followed by gel electrophoresis (Fig 2c) of total samples from both of the phases. (C) Non-enzymatic reactions were performed as described in Figure 2a in 25 mM Tris·HCl and 1 mM $MgCl_2$ for 1.5 hours. Total positive charge was 15 mM and total negative charge was 12.5 mM. Error bars indicate S.E.M from 3 independent experiments

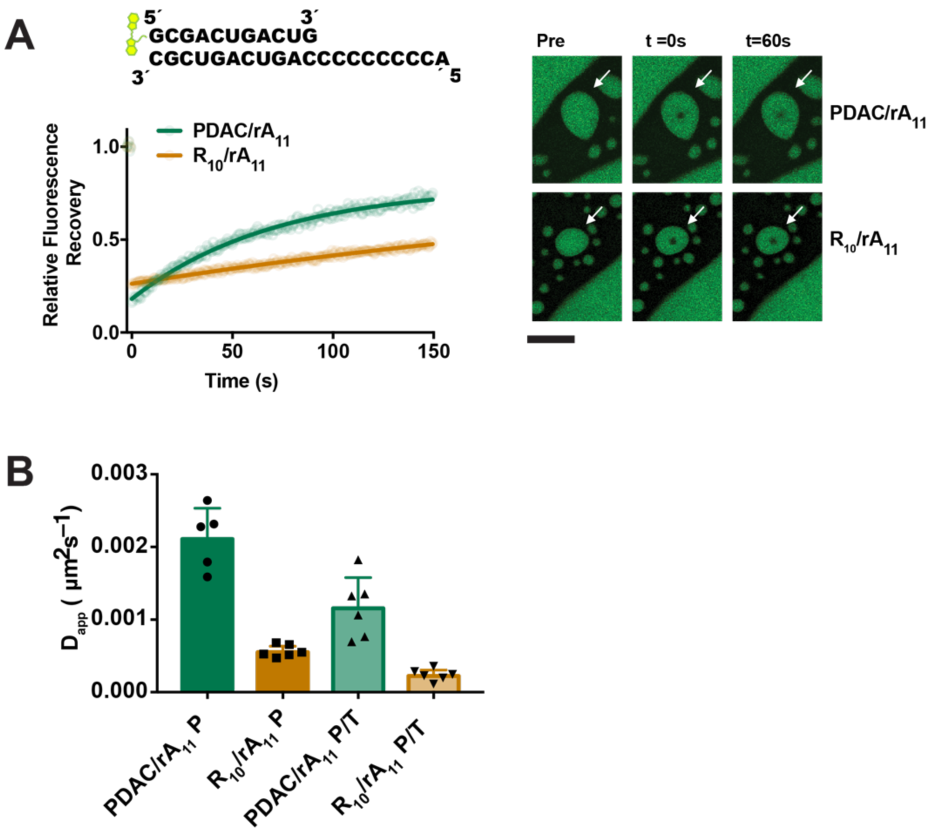

Supplementary Figure 5. Differential RNA diffusion in PDAC and R10 coacervates. (A) FRAP recovery curves for Cy-3 labeled RNA primer complexed with template in PDAC/rA11 ($t_{1/2}$ =53 ± 8 s) and R10/rA11($t_{1/2}$ = 286 ± 56 s) coacervates at 10 mM charge-balanced condition (These values are for single measurements, averages are reported in the text). The single fit from five independent experiments is plotted as a solid line. All the data points are shown in semi-transparent circles. Representative images of PDAC/rA11 (top) and R10/rA11 (bottom). Scale bar represents 5 µm. (B) Apparent diffusion coefficients calculated from the FRAP experiments for primer only (P) and primer-template complex (P/T). Error bars represent S.E.M (n=5)

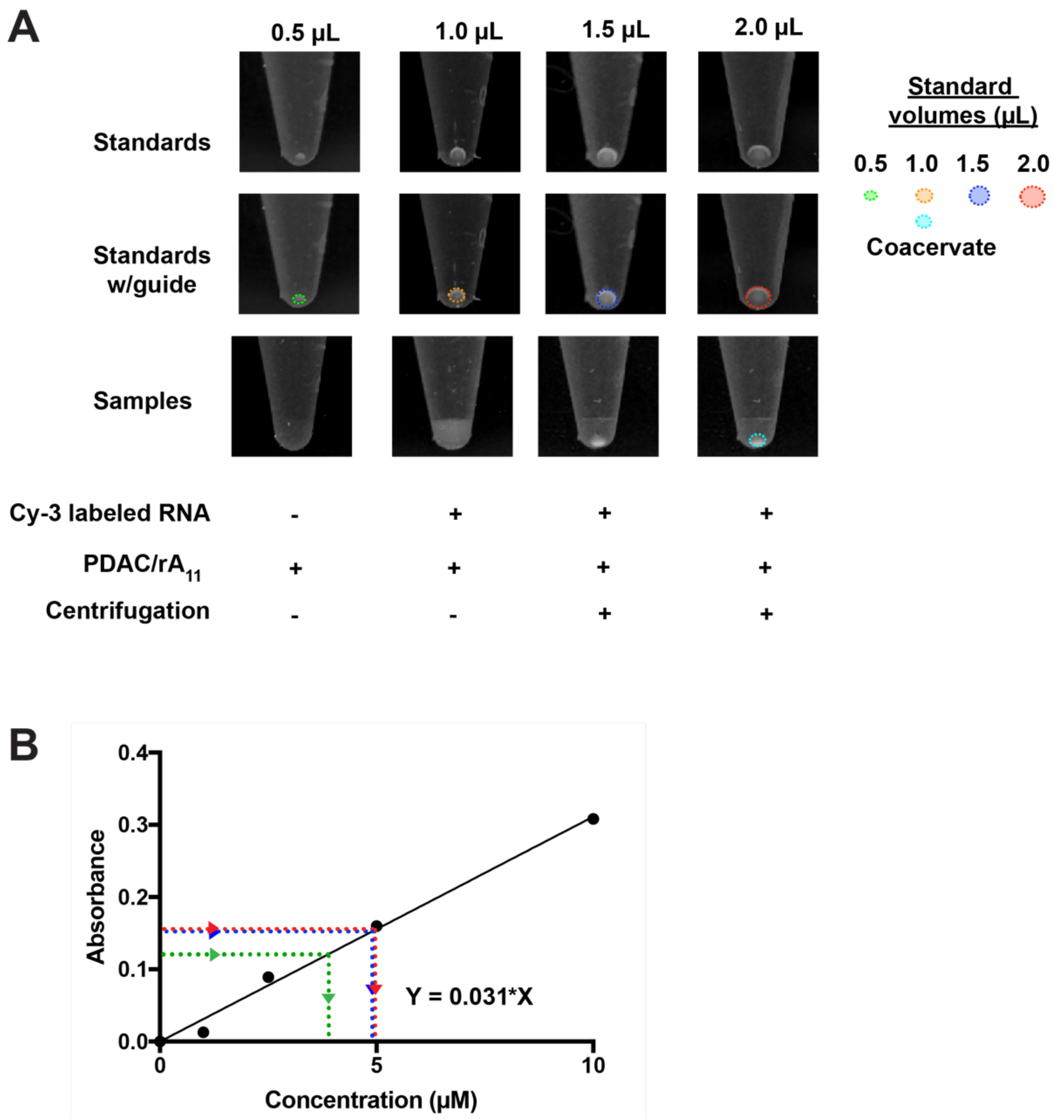

Supplementary Figure 6. Coacervate volume and magnesium levels. (A) 0.5 µL, 1 µL, 1.5 µL and 2.0 µL of 100 µM Cy-3 labeled RNA primer was added to tubes and were centrifuged. Tubes were then placed on a UV-screen and images were collected. Top panels show unmodified images and middle panels include outline of volume to guide the eye. To measure coacervate volumes, 20 µL of PDAC/rA11 coacervates were generated in 5 mM $MgCl_2$-containing buffer and 1 µL of 100 µM Cy3-labeled RNA. Once the coacervates were generated, samples were centrifuged and the volume of coacervate was estimated using the standard generated above (bottom panels). The volume of coacervate most closely resembled the volume of 1 µL (cyan and orange). (B) Standard curve to determine magnesium concentration. Magnesium chloride solutions at 1, 2.5, 5, and 10 µM were made in water. Absorbance were then read in a Shimadzu Atomic Absorption Spectrometer 7000. Dotted blue, red and green lines represent measured Mg^{2+} levels in the presence of buffer, in presence of PDAC and rA11 (no centrifugation), and PDAC and rA11 (with centrifugation). The R^2 has a value of 0.99.

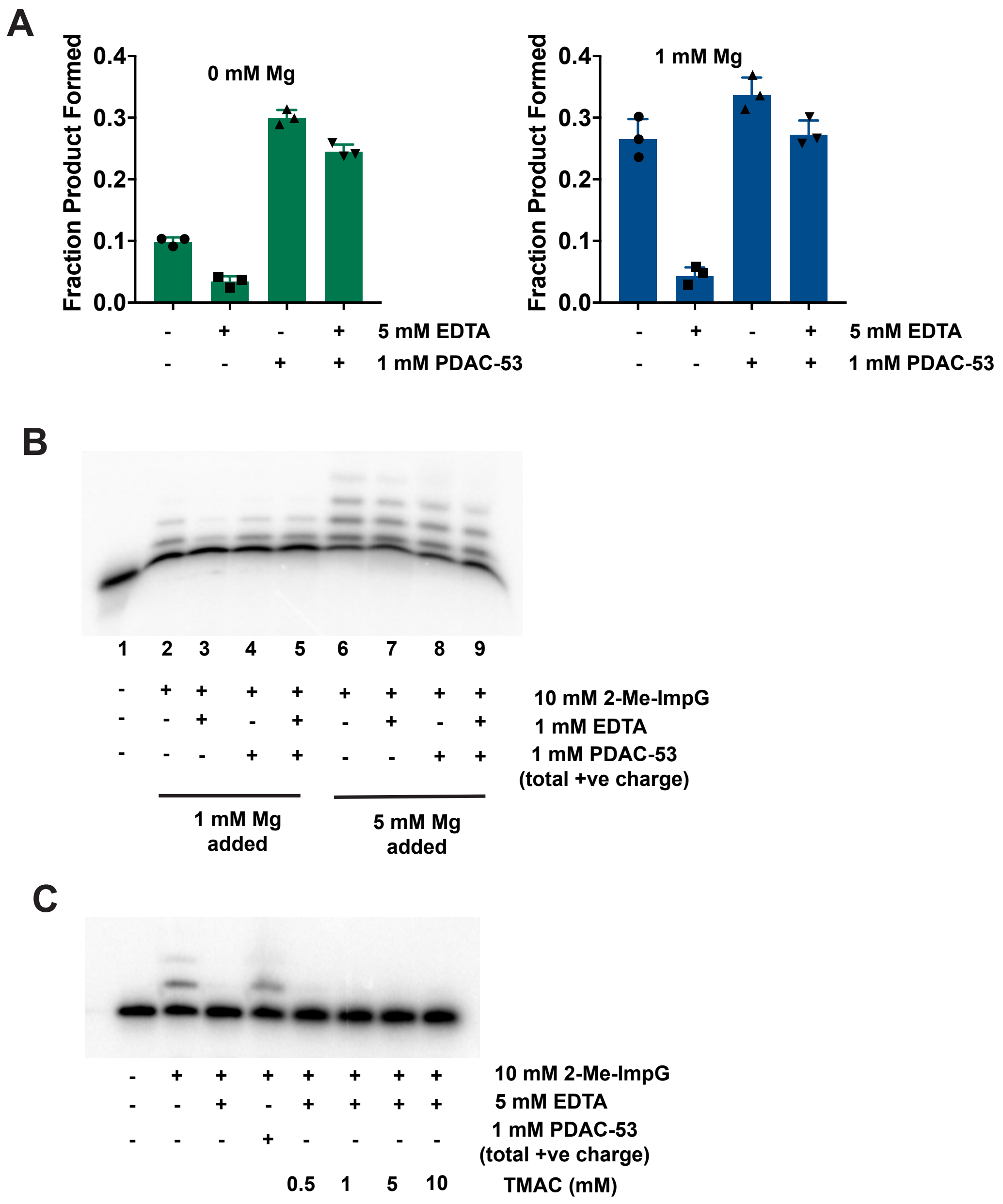

Supplementary Figure 7. PDAC-53 enhances non-enzymatic polymerization at sub-optimal Mg^{2+}. (A) Non-enzymatic polymerization reactions were performed for 1 h at room temperature. Reactions contained 25 mM Tris·HCl pH 8.0 with 10 mM 2-Me-ImpG and indicated levels of ions. Representative gel image is shown in Figure 3d. Error bars represent S.E.M (n=3) (B) Non-enzymatic polymerization reaction was assembled as described in Fig 3d. in the presence of the indicated levels of ions. (C) Non-enzymatic polymerization reactions were assembled as described in Fig 3d. in 1 mM $MgCl_2$. Indicated concentrations of tetramethylammonium chloride (TMAC) was added. Reactions were allowed to proceed for 3 hours at room temperature.

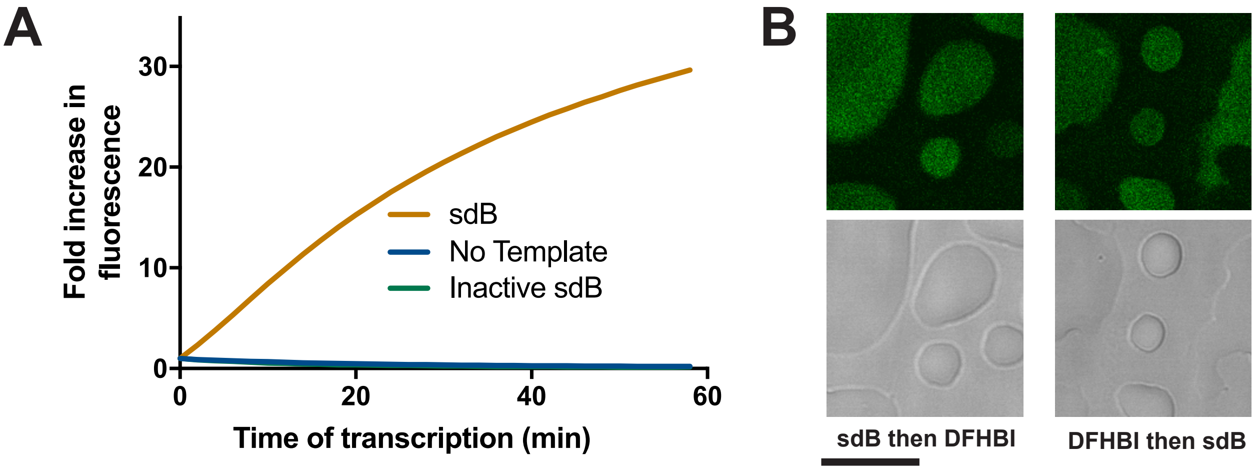

Supplementary Figure 8. RNA aptamer remains active inside PDAC/rA11 coacervates. (A) Transcription reactions were performed using 50 µM DFHBI. Fluorescence was measured using Applied Biosystems qPCR instrument (FAM-filter). Controls of no-template or inactive G63C/G87C mutant aptamer are provided. (B) Coacervate droplets exhibit fluorescence when coacervates are formed in the presence of pre-folded RNA followed by addition of DFHBI dye (left) or when coacervates were formed in the presence of DFHBI followed by the addition of pre-folded RNA (right). Coacervates contained 100 nM sdB RNA and 10 µM DFHBI in 25 mM Tris pH 8.0, 5 mM $MgCl_2$ and 5 mM KCl. 488 nm laser was used for excitation and emission window was 500-550 nm. Scale bar is 10 µm.

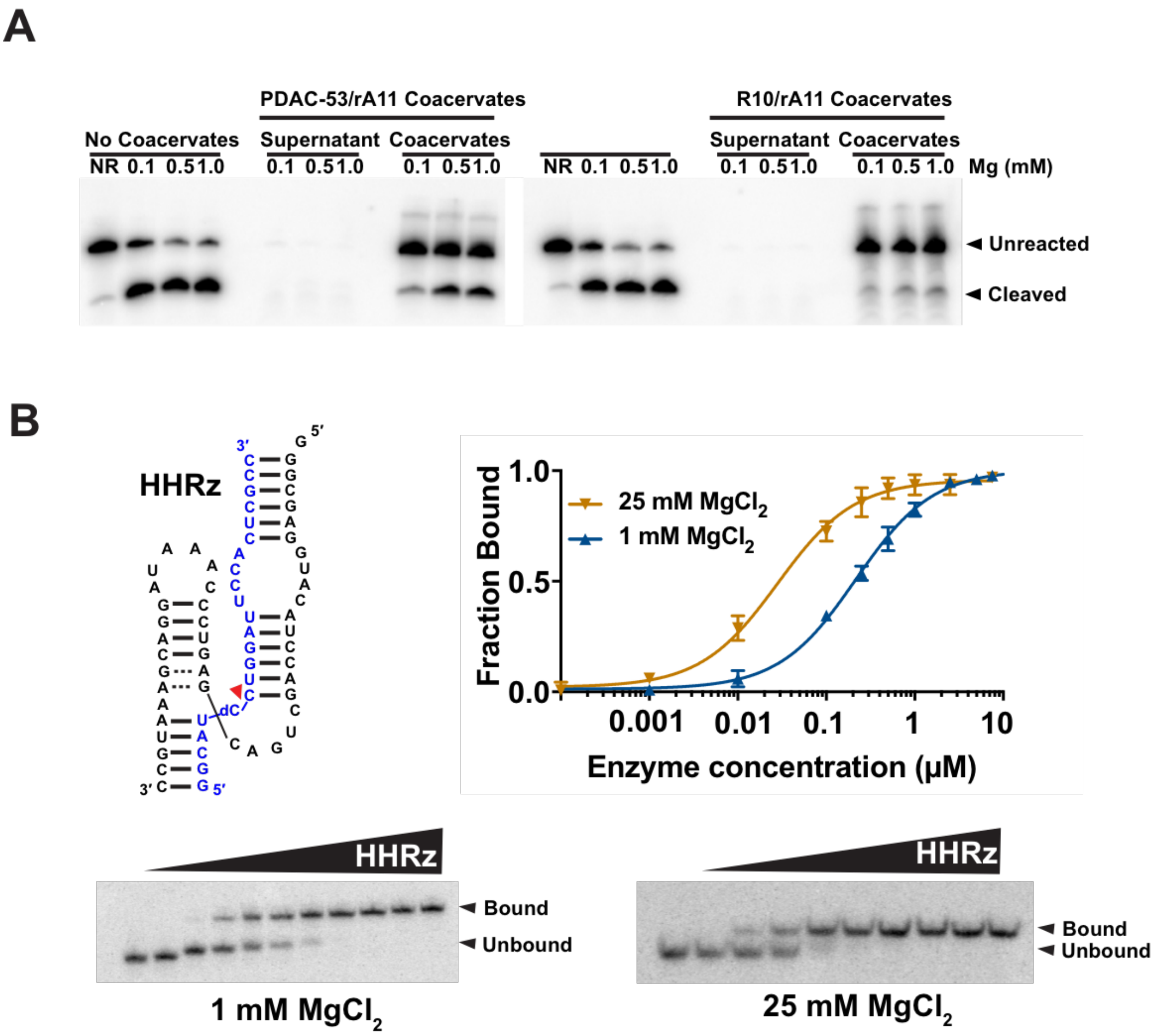

Supplementary Figure 9. Hammerhead ribozyme activity in coacervates. (A)Gel image showing the dilute supernatant phase of reactions described in Figure 5a. (B) Determination of apparent dissociation constant at high and low Mg^{2+}. (Left) Structure of the hammerhead ribozyme and the deoxysubstrate. C7 was changed to deoxy analog to prevent cleavage. (Right) Fraction substrate bound measured by electrophoretic mobility shift yields a dissociation constant of 220±10 nM and 28±3 nM at 1 mM and 25 mM $MgCl_2$, respectively, in the background of 2.5 mM KCl. Error bars represent standard error of mean from three independent experiments. Representative gel images used to generate the binding curve is shown in the bottom.

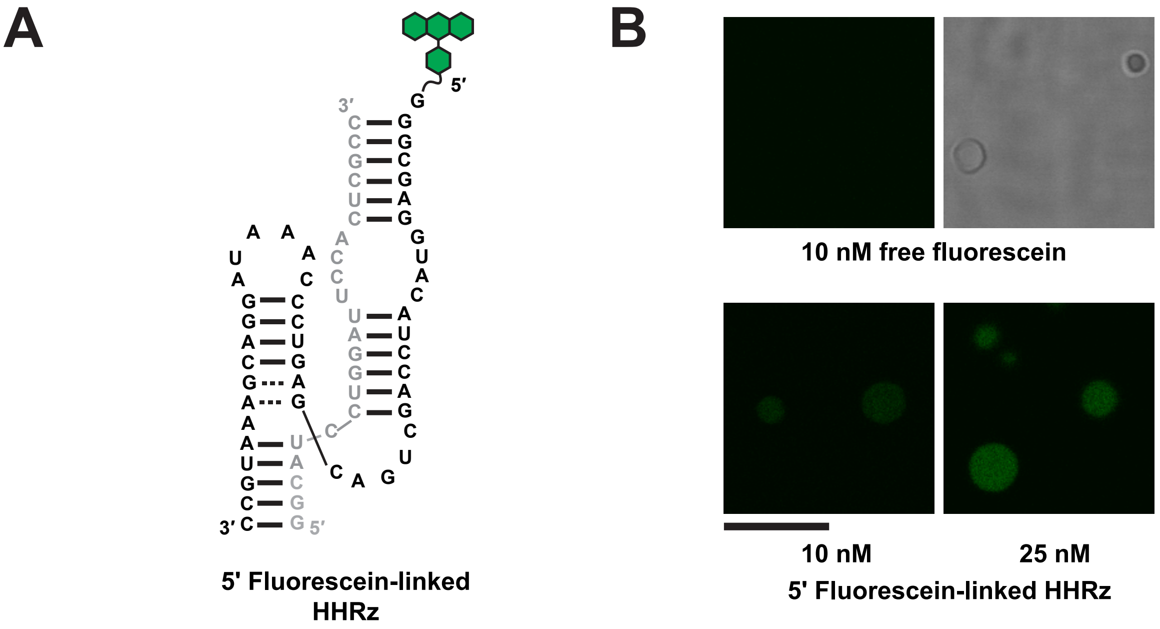

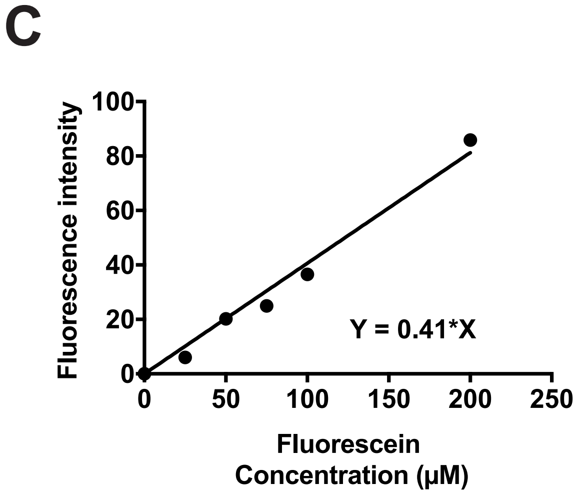

D

Concentration added (μM)	Concentration inside (μM)
0.01	44.7 ± 2.9
0.025	109.7 ± 5.0
0.05	140.4 ± 9.1

Supplementary Figure 10. PDAC/D_{10} coacervates concentrate hammerhead ribozyme. (A) Structure of the hammerhead ribozyme substrate complex where the enzyme strand is labeled with fluorescein. (B) Images of PDAC/D_{10} coacervates with 10 nM free fluorescein in fluorescent channel (top left) and DIC (top right). Fluorescent images of PDAC/D_{10} coacervates with fluorescein-linked hammerhead ribozyme (bottom panels) Scale bar= 10 µm. All coacervates were made in 25 mM Tris·HCl pH 8.0 and 1 mM $MgCl_2$. Concentration of fluorescein-labeled hammerhead ribozyme inside coacervates was estimated by comparing the fluorescence reading inside coacervates with the standards. Errors represent standard error of mean of at least three independent measurements. The R^2 has a value of 0.99.

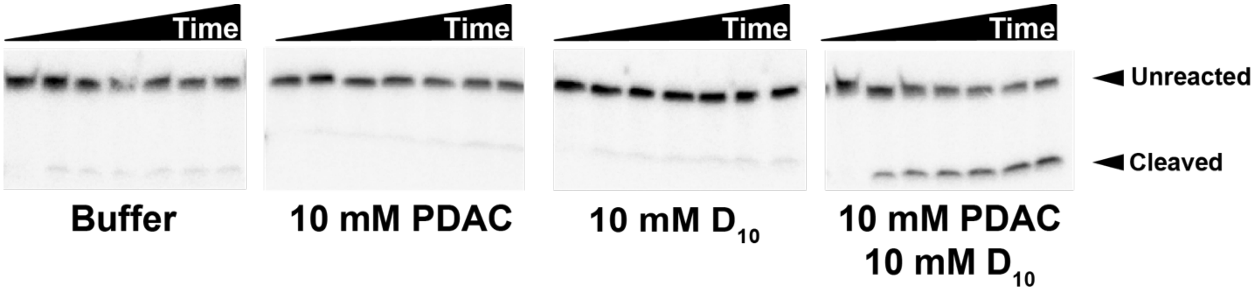

Supplementary Figure 11. Ribozyme enhancement requires coacervates. Gel images showing ribozyme cleavage in buffer, PDAC-53 alone, D10 alone, or in the presence of PDAC-53 and D10. Concentrations of polyions indicate the total charge from each of the polymers. Reactions contained 1 nM enzyme and 130 pM substrate in 25 mM Tris·HCl pH 8.0, 1 mM $MgCl_2$ and 2.5 mM KCl. Reactions were stopped after 5, 10, 15, 30, 60 and 120 min and separated by 15% denaturing PAGE.

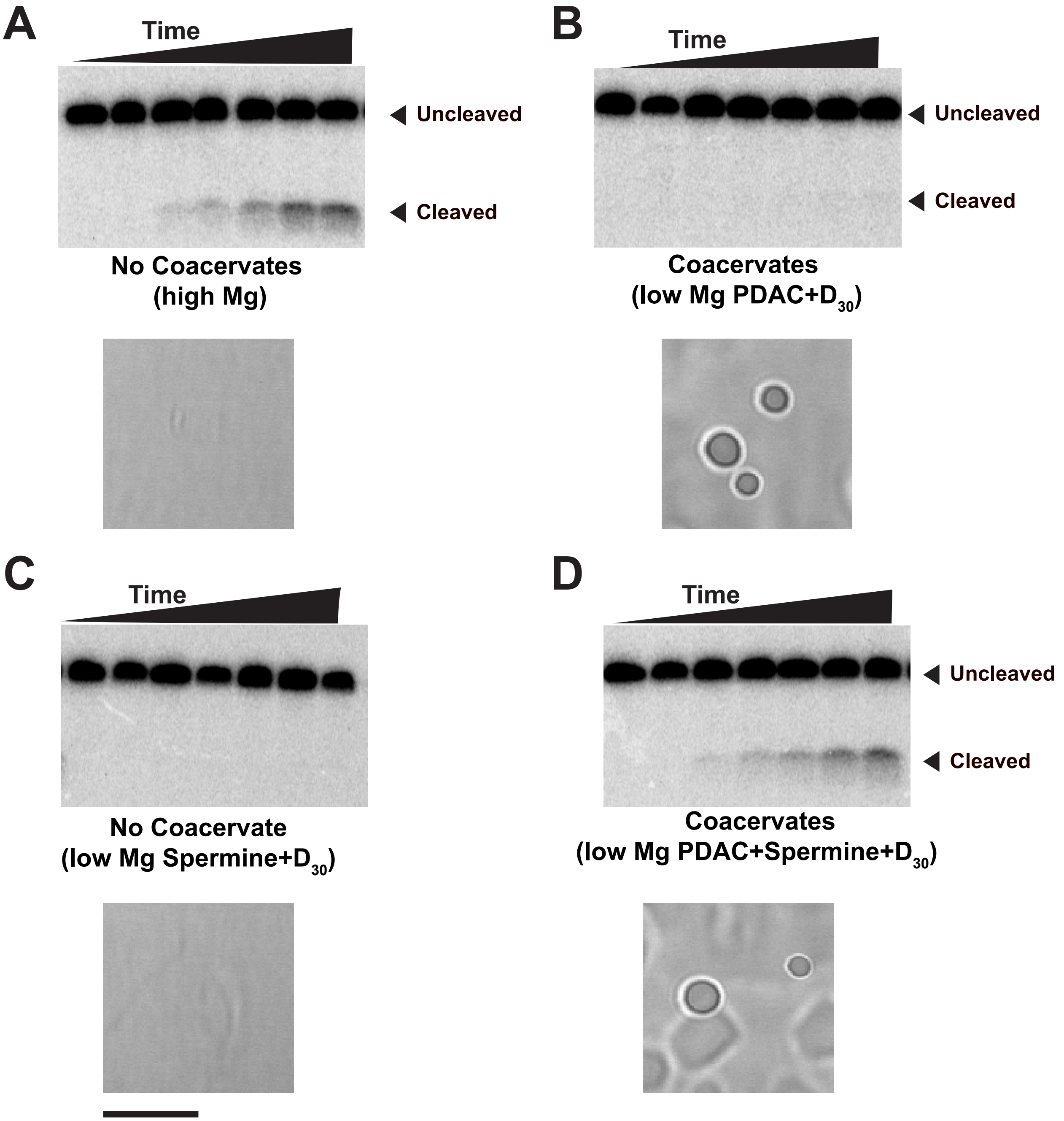

Supplementary Figure 12. Enhancement of hairpin ribozyme requires coacervates. (A) Ribozyme reactions were performed as described in Fig. 6b. Reaction (A) is shown in 6b, and was performed in 25 mM Tris·HCl pH 7.5, 25 mM $MgCl_2$, and 25 mM KCl. For (B-D) both $MgCl_2$ and KCl were reduced to 2.5 mM each. Reaction (D) is also shown in 6b. DIC images of solutions containing different polyions is shown below respective gel images. Scale bar= 10 µm.

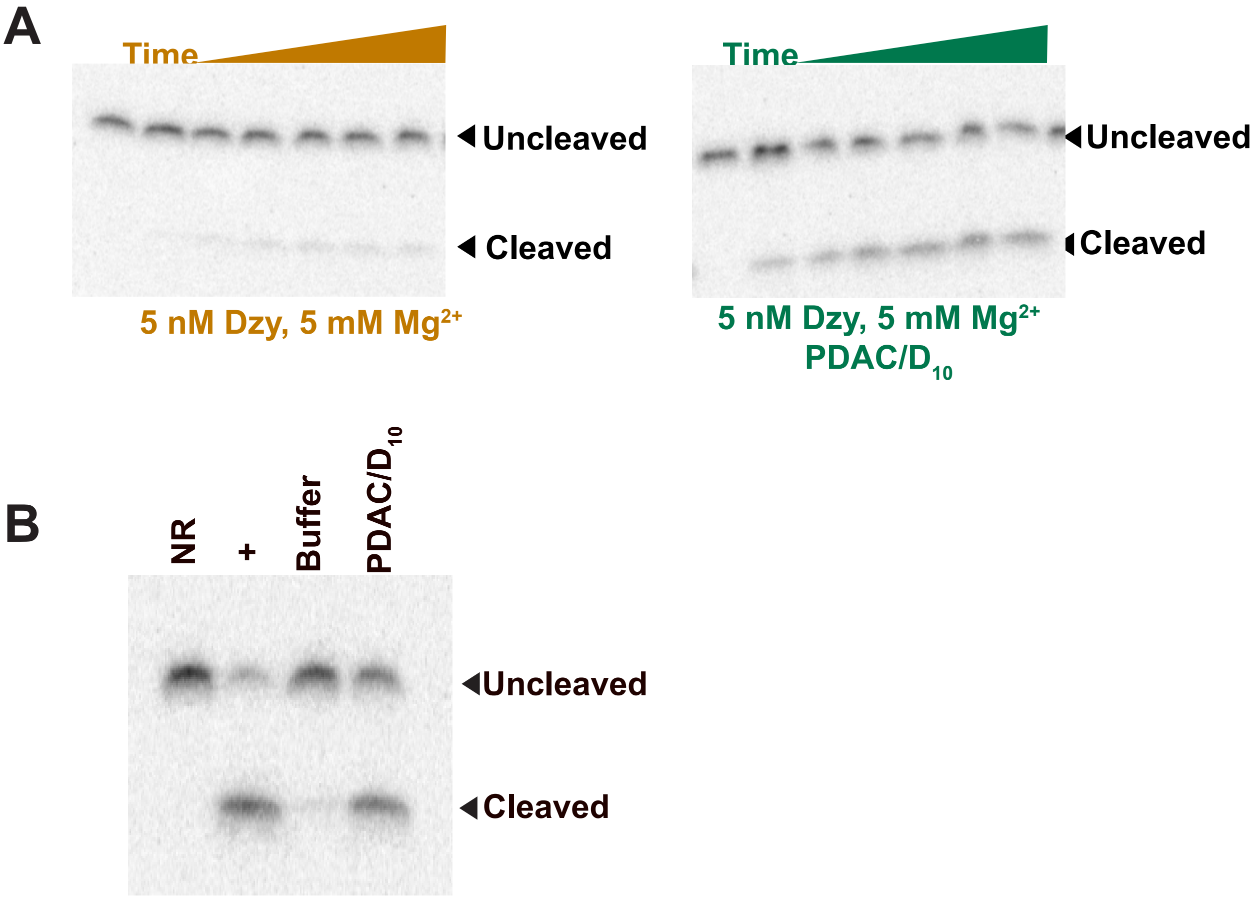

Supplementary Figure 13. Coacervate-mediated stimulation of a DNAzyme. (A) Reactions were performed as described in Fig. 7 b. except 5 mM $MgCl_2$ was used. (B) Samples after 2 hr reaction were loaded in adjacent lanes to show identical electrophoretic mobilty shift of the products. "NR" indicates the substrate alone, "+" indicates positive control with 1 μM of the enzyme strand and 0.25 pM of the substrate. All other lanes contain 5 nM enzyme and 0.25 pM substrate in 25 mM Tris·HCl pH 8.0, 2.5 mM $MgCl_2$ and 2.5 mM KCl.

A

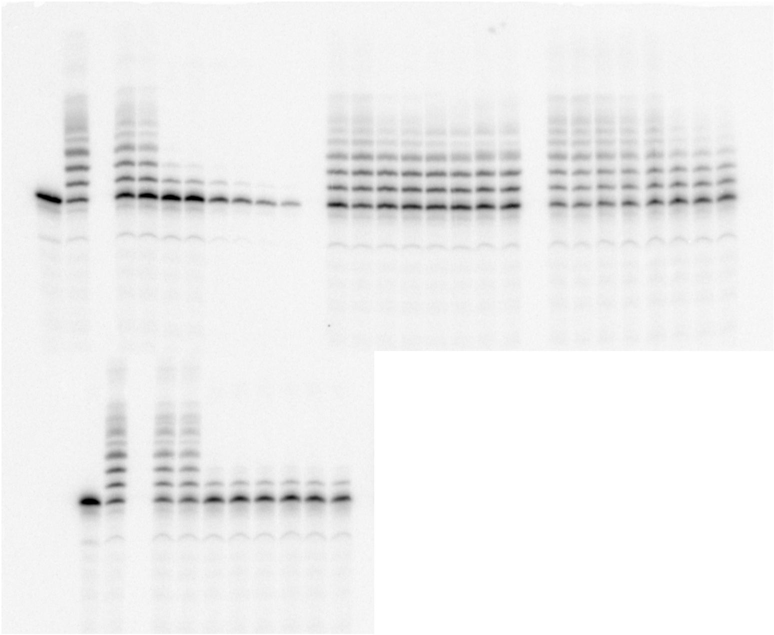

B

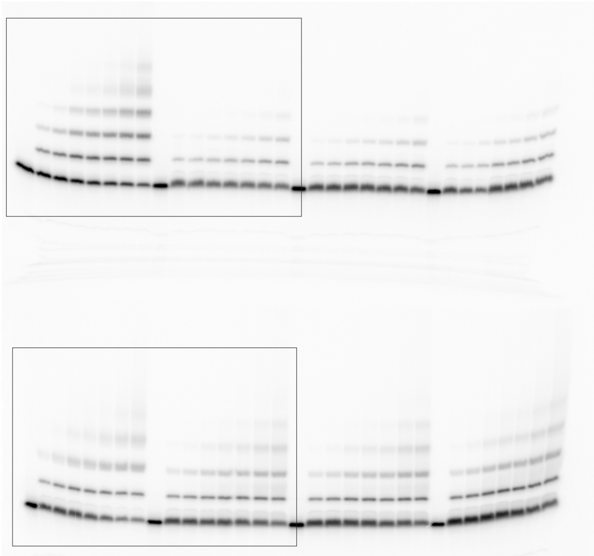

C

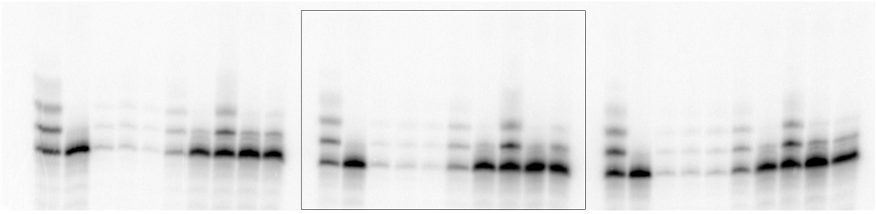

Supplementary Figure 14 Uncropped gel images shown in Figure 1c (A), Figure 2a (B) and Figure 2b (C). Black rectangle denotes the selection shown in the main text.

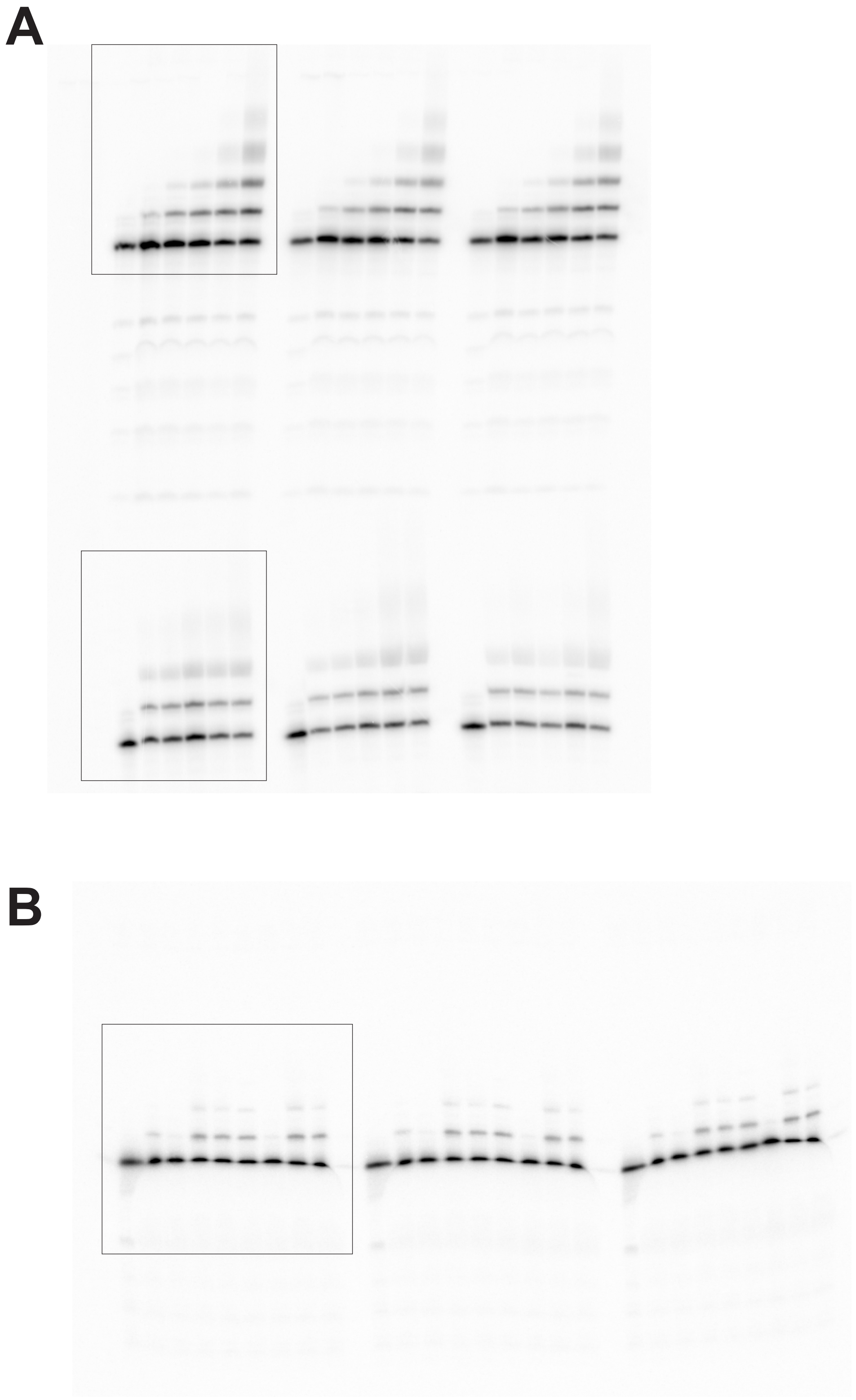

Supplementary Figure 15 Uncropped gel images shown in Figure 3b (A) and Figure 3d (B). Black rectangle denotes the selection shown in the main text.

A

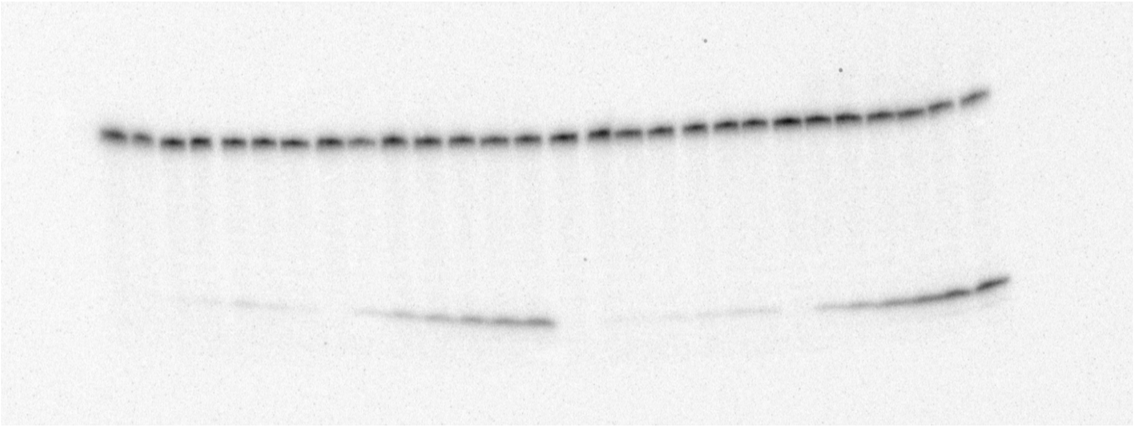

B

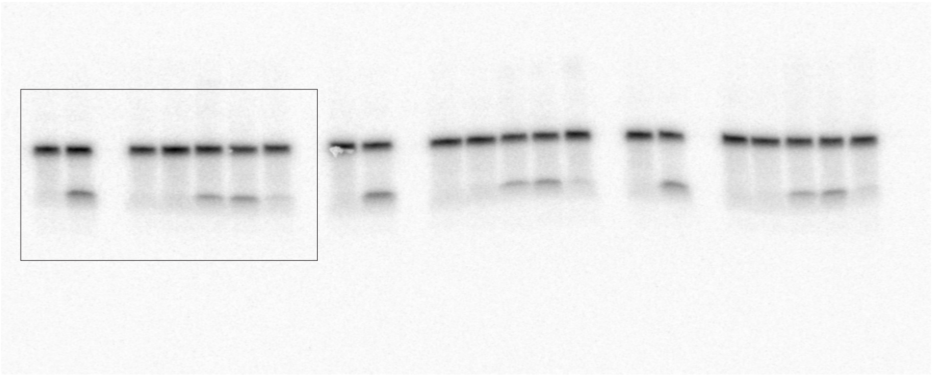

Supplementary Figure 16 Uncropped gel images shown in Figure 5c (A) and Figure 5d (B). Black rectangle denotes the selection shown in the main text.

A

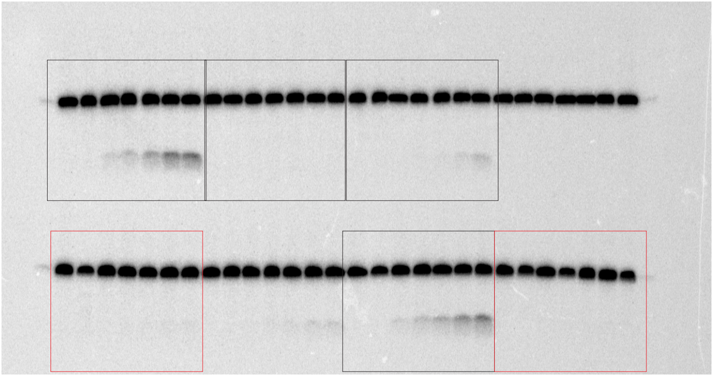

B

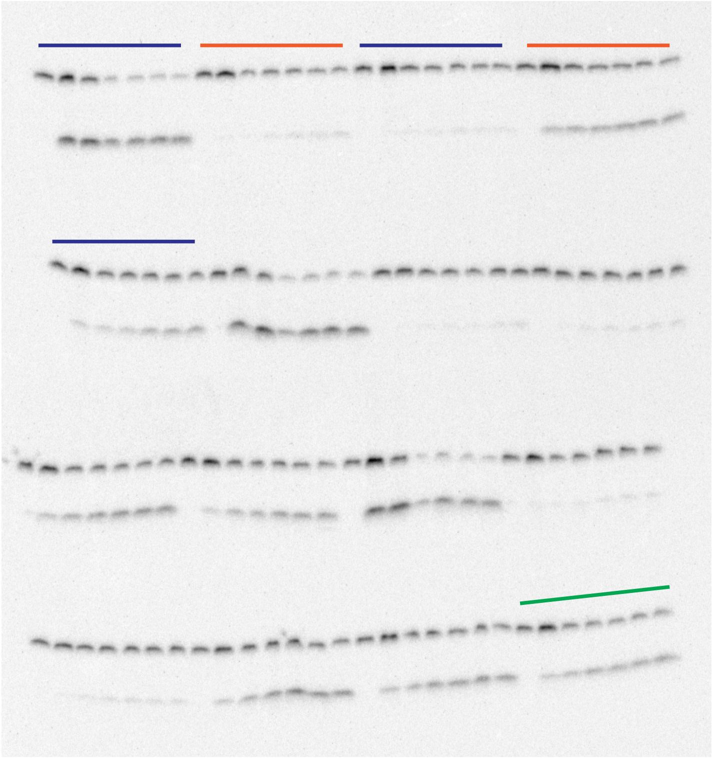

Supplementary Figure 17 Uncropped gel images shown in Figure 6a. and 6b and also in Supplementary Figure 12a and 12d (black squares) and Supplementary Figure 12b and c (red squares) (A) Figure 7a and b (blue lines) and Supplementary Figure 13a (orange lines). All other bands are replicates of these experiments. Green line was an unrelated experiment ran on the same gel (B).
