# Supplementary material for: Template-directed RNA polymerization and enhanced ribozyme catalysis inside membraneless compartments formed by coacervates
Source: Nat Commun. 2019 Jan 30;10:490. doi: 10.1038/s41467-019-08353-4 (PMC6353945; doi:10.1038/s41467-019-08353-4)
Supplement: Supplementary file 3 — Peer Review File [file 41467_2019_8353_MOESM3_ESM.pdf]

Reviewers' Comments:

Reviewer #1:
Remarks to the Author:
In this revised manuscript [REDACTED], Poudyal and coworkers have addressed my concerns and those of the three other reviewers from the prior submission. They have done so with significant changes to the text as well as the incorporation of new experiments. Of particular relevance to my comments, they have now demonstrated the utility of the coacervates studied in promoting the activity of a DNAzyme and another ribozyme. This was my principal sticking point with the earlier submission, and I thus now recommend publication in Nature Communications.

Reviewer #2:
Remarks to the Author:
I think the authors have sufficiently addressed my own and i think also the concerns of the other referees. This is now a very thorough and carefully exercised paper. I recommend publication.

Reviewer #3:
Remarks to the Author:
The authors have addressed the technical comments and I fully support publication in Nat. Commun.
